# Crush injury syndrome in earthquakes: a systematic review and meta-analysis on its frequency and complications

**DOI:** 10.1186/s12873-026-01516-9

**Published:** 2026-04-02

**Authors:** Parisa Rostami, Asghar Jafari Rouhi, Reyhaneh Hajebrahimi, Gholamreza Faridaalaee, Reza Mostafaei Yonjali, Kavous Shahsavarinia, Hanieh Salehi-Pourmehr

**Affiliations:** 1 Research Center for Evidence-Based Medicine, Faculty of Medicine, Tabriz University of Medical Sciences, Azadi Street- Golgasht Ave, Tabriz, East Azarbaijan, Tabriz, 51666/15731 Iran; 2 Emergency and Trauma Care Research Center, Tabriz University of Medical Sciences, Tabriz, Iran; 3 Student Research Committee, Tabriz University of Medical Sciences, Tabriz, Iran

**Keywords:** Crush syndrome, Crush injury, Compartment syndrome, Rhabdomyolysis, Systematic Review

## Abstract

**Background:**

Crush syndrome is a life-threatening systemic complication of traumatic rhabdomyolysis and a leading cause of morbidity and mortality following earthquakes. Despite its clinical significance, a comprehensive synthesis of its global epidemiology, management, and outcomes is lacking. This systematic review and meta-analysis aimed to consolidate the evidence on CS in earthquake victims.

**Methods:**

We conducted a systematic review in accordance with PRISMA guidelines (PROSPERO: CRD420251177534). Electronic databases (PubMed/MEDLINE, Web of Science, CINAHL) were searched from inception through January 2024 for observational studies of earthquake casualties with CS. Outcomes included mortality, dialysis requirement, acute kidney injury (AKI), and other complications. Data were pooled using random-effects meta-analysis.

**Results:**

Fifty studies (1988–2023), primarily from Turkey, Iran, and China, were included. Substantial heterogeneity was observed, largely due to inconsistent definitions of CS. The pooled proportion of patients requiring dialysis was 0.49 (95% CI 0.40–0.58; I²=96.95%, 38 studies). The overall pooled mortality was 0.08 (95% CI 0.06–0.10; I²=93.0%, 39 studies), with significant regional variation (1% to 26%). AKI was the most frequent complication (pooled proportion 0.49; 95% CI 0.38–0.59). Marked creatine kinase elevation and metabolic derangements were consistently reported.

**Conclusions:**

CS following earthquakes carries a high burden of renal failure, with nearly half of affected patients requiring dialysis. Mortality is significant and influenced by geographic context and response capabilities. The profound heterogeneity in definitions and reported outcomes underscores an urgent need for standardized diagnostic criteria. Preparedness planning must prioritize early volume resuscitation, surge capacity for renal replacement therapy, and the development of context-adapted, evidence-based clinical guidelines.

**Supplementary Information:**

The online version contains supplementary material available at 10.1186/s12873-026-01516-9.

## Introduction

Earthquakes represent one of the most catastrophic natural hazards globally, imposing a profound and recurring burden on human populations across centuries [1]. Historical records document at least 8.3 million fatalities attributable to seismic events across 117 countries from 856 BC to 2022 [2]. The mortality distribution is geographically heterogeneous, with densely populated, high-risk regions such as China, Pakistan, and Iran accounting for a significant proportion of deaths in recent decades [3]. When normalized by population size, metrics such as the Earthquake Fatality Load (EQFL) further identify nations, including Ecuador, Haiti, Iran, Lebanon, Portugal, and Turkmenistan, as having endured the highest relative mortality burdens over the past five centuries [4]. These patterns underscore the persistent and inequitable global impact of seismic disasters. Beyond immediate trauma, earthquakes are a predominant cause of large-scale crush injuries due to structural collapse and prolonged entrapment under debris. Crush syndrome, a systemic manifestation of reperfusion injury following compressive trauma, is among the most serious and debilitating sequelae, complicating an estimated 2–15% of earthquake-related injuries [5, 6]. Its incidence varies with seismic magnitude and rescue efficiency, having affected over half of hospitalized patients after the 1995 Kobe earthquake and the majority of ICU admissions following the 1999 Marmara earthquake [7, 8]. The release of intracellular contents, including myoglobin, potassium, and phosphate, from ischemic muscle upon reperfusion initiates the pathophysiological cascade. This leads to characteristic laboratory findings: marked elevation of creatine kinase (CK), hyperkalemia, hyperphosphatemia, hypocalcemia, metabolic acidosis, and myoglobinuria [9, 10]. These biochemical disturbances underline life-threatening complications such as hypovolemic shock, acute kidney injury (AKI), cardiac arrhythmia, disseminated intravascular coagulation [1], and sepsis [11, 12]. Myoglobin-induced oxidative stress and tubular obstruction, compounded by renal hypoperfusion from third-spacing of fluids, are central to the development of AKI, a leading cause of mortality in this context [13, 14]. Although established management principles, including aggressive early fluid resuscitation, timely renal replacement therapy, and judicious fasciotomy, can improve outcomes, their implementation in disaster settings is severely constrained. Logistical challenges such as delayed extrication, disrupted infrastructure, resource limitations, and personnel shortages often compromise care, highlighting the critical need for effective triage, early diagnosis, and context-adapted management protocols [15–17]. While existing studies describe clinical manifestations and treatment approaches for CS following earthquakes, a comprehensive, integrative synthesis of global epidemiological trends, clinical presentations, therapeutic interventions, and patient outcomes is lacking. Such a synthesis is essential for informing evidence-based preparedness planning, optimizing resource allocation, and guiding the development of standardized clinical guidelines for mass-casualty seismic events.

## Aim of the review

To address this gap, the present review will systematically consolidate the available literature on crush injury syndrome in earthquake victims, with a focus on epidemiology, clinical and laboratory characteristics, management strategies, and outcomes. Specifically, this review aims to:

1. Quantify the mortality rate and major sequelae of CS following earthquakes.
2. Synthesize reported laboratory data, particularly markers of muscle injury and renal dysfunction.
3. Evaluate the incidence of acute kidney injury and requirements for renal replacement therapy (dialysis).

## Materials and methods

### Protocol registration and review design

This systematic review was conducted in accordance with the Preferred Reporting Items for Systematic Reviews and Meta-Analyses (PRISMA) guidelines. The protocol was registered prospectively with PROSPERO (Registration ID: CRD420251177534).

### Eligibility criteria

Studies were selected based on the following PICOS framework:

Population: Human victims of any earthquake globally, diagnosed with crush injury or CS.

Exposure: Crush injury sustained during an earthquake.

Outcomes: Primary: mortality rate and dialysis requirement. Secondary: incidence/prevalence of complications (e.g., AKI, hyperkalemia, sepsis, ARDS, compartment syndrome) and other relevant clinical outcomes.

Study Designs: Observational studies, including cohort (prospective and retrospective), case-control, and cross-sectional studies, as well as large case series (≥ 10 patients). Case reports, editorials, commentaries, and non-English publications were excluded.

### Search strategy

A comprehensive, three-step search strategy was employed to identify all relevant literature.

1. An initial limited search in PubMed/MEDLINE was performed, followed by an analysis of text words in the titles and abstracts of relevant papers and of the index terms used to describe articles.
2. A formal search using all identified keywords and controlled vocabulary (e.g., MeSH terms) was then conducted across the following electronic databases from inception through January 2024: PubMed/MEDLINE, Web of Science (Core Collection), and CINAHL via the EBSCOhost platform (encompassing the Virginia Henderson Global Nursing e-Repository). Google Scholar was also searched to identify grey literature and ensure breadth.
3. The reference lists of all included articles were screened for additional eligible studies.
4. The search strategy combined terms related to: (1) earthquakes (e.g., “seismic,” “natural disaster”), (2) crush injury (e.g., “Crush Syundrome,” “rhabdomyolysis,” “compartment syndrome”), and (3) outcomes (e.g., “mortality,” “renal replacement therapy,” “dialysis,” “acute kidney injury”).

No language restrictions were applied during the initial electronic database searches. Non-English studies were excluded during full-text screening due to feasibility constraints. Full search strategies and retrieval results for PubMed/MEDLINE, Web of Science, and CINAHL are provided in Supplementary Files 1.

### Study selection

Identified citations were collected and uploaded into EndNote X9 (Clarivate Analytics) for deduplication. Titles and abstracts were screened independently by two reviewers (initials blinded for review) against the inclusion criteria. Potentially relevant studies were retrieved in full text and assessed in detail by the same two independent reviewers. Disagreements were resolved through discussion or by consultation with a third reviewer. The selection process was documented using a PRISMA flow diagram.

### Data extraction

Quantitative data were extracted from included studies by two independent reviewers using a pre-piloted, standardized data extraction form. The extracted information included:

1. Study characteristics: author, publication year, country, study design, earthquake event, and time frame.
2. Population characteristics: total sample size, number of crush injury patients, age, sex.
3. Outcome data: mortality rate, incidence of AKI, dialysis requirement, prevalence of key complications (e.g., hyperkalemia, sepsis, fasciotomy), and relevant laboratory parameters (e.g., peak creatine kinase).

### Assessment of methodological quality

The methodological quality of included studies was critically appraised independently by two reviewers using the Joanna Briggs Institute (JBI) critical appraisal checklists appropriate for each study design. Discrepancies in appraisal were resolved by consensus. Studies were not excluded based on quality score alone; however, the findings of the quality assessment were used to inform the interpretation of results and the strength of conclusions during narrative synthesis and in the discussion of limitations. All of the studies were retrospective and, therefore, categorized as low-quality studies.

### Data synthesis

Extracted data were analyzed using Comprehensive Meta-Analysis software (version 2.2; Biostat, Englewood, NJ). Pooled estimates were calculated using a random-effects model based on the DerSimonian–Laird method to estimate between-study variance (τ²). Results are presented as proportions with 95% confidence intervals (CIs). This approach was selected a priori to account for anticipated clinical and methodological heterogeneity. Statistical heterogeneity was assessed using the I² statistic and the Cochran’s Q chi-square test. An I² value > 50% was considered to represent substantial heterogeneity. Where statistical pooling was inappropriate due to significant clinical or methodological heterogeneity, a narrative synthesis was performed. Findings were presented in text and summarized in structured tables and figures, grouped by outcome or earthquake event.

## Results

### Study selection

**Fig. 1:**
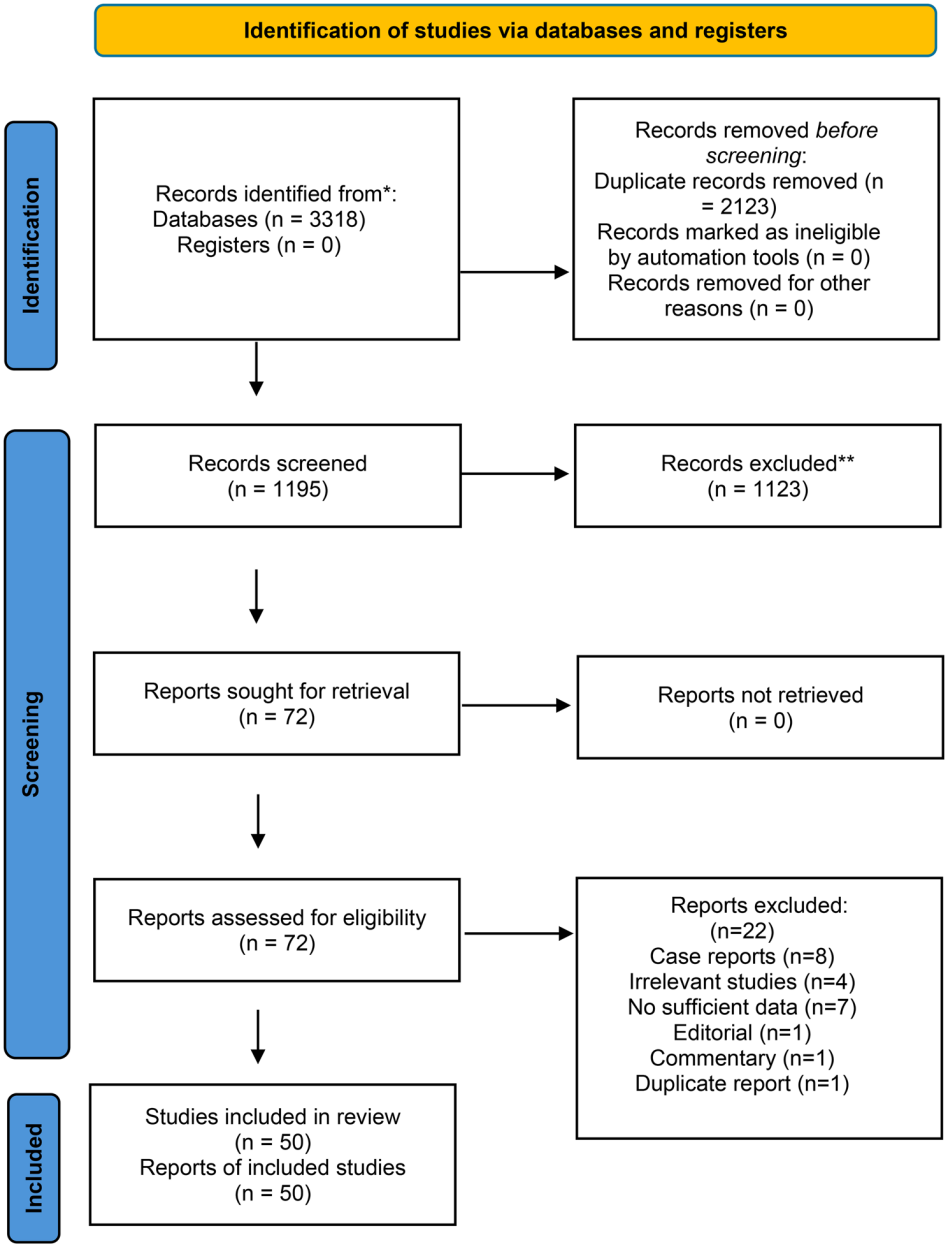
The PRISMA flow diagram shows the search and screening processes

The systematic search identified 3,318 records published through January 2024. After removing 2,123 duplicates, 1,195 unique records underwent title and abstract screening. This screening excluded 1,123 records that were unrelated to earthquake-related crush injury (e.g., focused on other disaster types or non-traumatic rhabdomyolysis). The remaining 72 full-text articles were assessed for eligibility, of which 22 were excluded for reasons (e.g., insufficient outcome data, irrelevant patient population). Ultimately, 50 studies met all inclusion criteria and were incorporated into the systematic review. The study selection process is detailed in the PRISMA flow diagram (Fig. 1).

### Study characteristics

The 50 included studies encompassed data from earthquakes across 11 countries, spanning from 1988 to 2023. Studies from Turkey were the most frequent (*n* = 28, 56%), primarily investigating the 1999 Marmara and 2023 Kahramanmaraş earthquakes. Six studies (12%) originated from Iran (mainly the 2003 Bam earthquake), and another six from China (primarily the 2008 Wenchuan earthquake). The vast majority of studies employed a retrospective cohort or case series design (*n* = 44, 88%); the remainder included case-control, descriptive, and cross-sectional designs (*n* = 6, 12%). A summary of included studies is presented in Tables 1 and 2, and Table 3.

**Table 1:** Characteristics of the included studies

Authors (Year)	Country / Setting	Study Design	Sample Size	Male/Female	Definition of CS	Diagnosis Criteria Used	Time to Presentation
Adachi et al. (1998) [7]	Japan/Kobe Earthquake	Case-Control	Case: 29/Control:10	total: 64% Male, 36% Female/ Case: 58.6% Male, 41.4% Female/ Control: 80% Male, 20% Female	crushed pelvis or leg, with or without fracture	Group A: BUN > 21 mg/dl and Cr > 1.5 mg/dl, with swelling of injured limb(s) and renal failure/Group B: the remaining patients	Admitted on the day of or the day after the earthquake
Akbaba et al. (2023) [51]	Turkey/Kahramanmaraş Earthquake	Retrospective observational	252 (54 rescued from the rubble), (182 affected by environmental conditions of disaster), (16 children with chronic illnesses), 34 out of 52 rescued children have compartment syndrome	49.2% Male, 50.8% Female	direct injury due to the collapsing material and debris	CS: systemic symptoms (local tissue injury, organ dysfunction including reduced urinary output), and metabolic abnormalities (acidosis, hyperkalemia, hypocalcemia)	6 h (in dialysis group), 12 h (in non-dialysis group)
Akgun et al. (2023) [52]	Turkey/Kahramanmaraş Earthquake	Retrospective observational	116	60.3% Male, 39.7% Females	pallor, severe pain, paresthesia, pulselessness, and paralysis	SSI: a positive intraoperative culture at the surgical site during any debridement within 30 days after fasciotomy, pathology samples of microbiological pathogens, and reoperation due to infection, Acute Compartment Syndrome: Diagnosed based on clinical presentation	fasciotomy time: 25.5 h (SSI group), 24 h (non-SSI group)
Akkoç et al. (2024) [53]	Turkey/Kahramanmaraş Earthquake	Retrospective cross‑sectional	38	31.6% Male, 68.4% Female	systemic consequence of muscle tissue damage (rhabdomyolysis) caused by pressure, which leads to the release of toxic muscle cell components into the extracellular fluid	NM	variable; examples include 2 h, 8 h, and 9 h based on individual case
Aoki et al. (2006) [49]	Japan/Kobe Earthquake	Retrospective cohort	345	Not specified	patients had sustained injuries by being compressed under collapsed buildings, and patients manifested swelling or neurologic disturbances at the affected anatomic site	crush injury without systemic syndrome, moderate CS marked by renal failure, severe CS requiring hemodialysis, and fatal CS	NM
Asfuroğlu et al. (2023) [54]	Turkey/Kahramanmaraş Earthquake	Retrospective	204 (66 crush injury)	48% Male, 52% Female	crush injury with or without fracture		First patient ~17:30 on Day 1; admissions intensified from Day 2
Atef et al. (1994) [26]	Iran/Manjil-Rudbar Earthquake	Descriptive	495	NM	ARF (AKI) due to crush injury	ARF (AKI) requiring dialysis, elevated muscle enzymes/K/P, abnormal urinalysis, compartment syndrome signs, oligoanuria/elevated creatinine.	admission to hospital: 14 h
Aydin et al. (2024) [37]	Turkey/Kahramanmaraş Earthquake	Retrospective cross-sectional	62 (60 CS)	54.8% Male, 45.2% Female	systemic organ dysfunction, such as acute renal failure	systemic organ dysfunction (e.g., AKI) following crush injury	30.5 h
Bakkaloglu et al. (2024) [55]	Turkey/Kahramanmaraş Earthquake	Descriptive Study	903	51% Male, 49% Female	Crush injury: direct trauma of a body part, such as extremities, with manifestations of local findings, CK >5x ULN (upper limit nomal)	Crush-AKI: Crush injury + ≥1 nephrological abnormality (oligo/anuria, serum creatinine >2x ULN, potassium >6 mmol/l, or uric acid >8 mg/dl)	13 h, 15 (AKI group), 8 (non-AKI group), 12 (in dialysis group), 9 (in non-dialysis group)
Çağıran et al. (2022) [56]	Turkey/Aegean Sea Earthquake	Retrospective Analysis	152 (6 CS)	39.5% Male, 60.5% Female	damage to the kidneys caused by myoglobin arising from the muscle tissue	NM	7.37 ± 8.02 h (mortal), 9.64 ± 17.7 h (survivors). Total mean: 9.05±15.79 h
Xiaolei et al. (2010) [27]	China/Wenchuan Earthquake	Retrospective observational	58 CS	60.35% Male, 39.65% Female	presence of crush injuries (muscle injury from prolonged limb compression) and myoglobinuria with acute impairment of renal function	at least one of the following: urinary output < 400 ml/day AND/OR blood urea nitrogen (BUN) > 40 mg/dl, serum creatinine (Cr) > 2.0 mg/dl, uric acid (UA) > 8.0 mg/dl, potassium (K) > 6.0 mEq/l, phosphorus (P) > 8.0 mg/dl, bicarbonate < 15 mEq/l, total calcium (Ca) < 8.0 mg/dl	23.7 ± 19.3 h ADDIN EN.CITE (1)
Del Papa et al. (2019) [57]	Italy/L’Aquila Earthquake	Retrospective observational, Descriptive	171	36% Male, 64% Female	as resulting in death by asphyxiation, bleeding, and acute kidney injury (AKI).	specific lab or clinical criteria for CS diagnosis were not detailed as the study focuses on broader injury patterns	All included patients admitted within 96 h post-mainshock
Demir et al. (2024) [42]	Turkey/Kahramanmaraş Earthquake	Retrospective observational	36 (22 patients CS, 7 compartment syndrome)	30.6% Male, 69.4% Female	hemodynamic and metabolic disorders and acute renal failure following muscle injury due to prolonged compression of a limb	NM	14.94 ± 18.62 h
Demirkiran et al. (2002) [8]	Turkey/Marmara earthquake	Retrospective observational	18 crush injury patients	61.1% Male, 38.9% Female	traumatic rhabdomyolysis after prolonged continuous pressure, characterized by systemic involvement	presence of swollen limbs and history of limb compression	24.10 ± 22.24 h
Derici et al. (2002) [38]	Turkey/Marmara earthquake	Case-Control	34 (17 CS patients, 7 non-crush injury patients, 10 healthy controls)	76.5% Male, 23.5% Female	extensive muscle damage leading to acute renal failure	injuries from being crushed under collapsed building, swelling/neurological disturbances (motor/sensory deficits), peak creatine kinase (CPK) > 3000 U/L, abnormal urine (myoglobinuria/hematuria)	15 h ADDIN EN.CITE (1)
Döven S et al. (2024) [28]	Turkey/Kahramanmaraş Earthquake	Retrospective Analysis	649 pediatric earthquake victims (104 hospitalized) (59 CS, 17 compartment syndrome, 585 non-CS)	54.2% Male, 45.8% Female (hospitalized patients)	a systemic manifestation of rhabdomyolysis from sustained muscle pressure, potentially causing hypovolemic shock, AKI, compartment syndrome, electrolyte imbalances, and metabolic acidosis	“Crush Injury: Identified by limb compression and associated swelling CS: Diagnosed when crush injury is accompanied by myoglobinuria/hematuria, kidney failure, and peak creatine kinase (CPK) exceeding 1000 IU/l	4 h
Dönmez et al. (2001) [39]	Turkey/Marmara earthquake	Clinical and laboratory evaluation (case series)	20 CS	35% Male, 65% Female	traumatic rhabdomyolysis with systemic and local complications, characterized by hypovolemic shock, hyperkalemia, acute renal failure (ARF), and muscle necrosis	crushing injury to a large skeletal muscle mass, sensory/motor disturbances in compressed limbs, becoming tense/swollen, myoglobinuria and/or hematuria, Peak creatine kinase (CPK) > 1000 IU/l	17.9 ± 5.1 h
Ellidokuz et al. (2005) [58]	Turkey/Afyon Earthquake	cross-sectional	812	50% Male, 50% Female	NM	there are no detailed diagnostic criteria for CS in this study	Study conducted three months after the disaster.
Ensari e al. (2002) [59]	Turkey/Marmara Earthquake		38 crush injury (27 CS)	55.5% Male, 44.5% Female	the presence of swollen limbs with a history of limb compression	myoglobinuria, oligoanuria (urine output <20 ml/h), high serum urea nitrogen (>40 mg/dl) and creatinine (>2 mg/dl)	13.6 ± 2.16 h (in dialysis group), 11.53 ± 1.31 h (in non-dialysis group)
Erek et al. (2002) [45]	Turkey/Marmara Earthquake	Retrospective observational	639	54.5% Male, 45.5% Female	NM	NM	NM
Ersoy et al. (2002) ADDIN EN.CITE [46]	Turkey/Marmara Earthquake	Retrospective	60 CSa and dialysis patients	56.7% Male, 43.3% Female	traumatic rhabdomyolysis with systemic and local complications	NM	9.7 ± 2.0 h (survivors), 8.2 ± 1.4 h (non-survivors)
Fan et al. (2010) [60]	China/Wenchuan Earthquake		1038	45.9% Male, 54.1% Female	NM	NM	NM
Hu et al. (2010) [61]	China/Wenchuan Earthquake	Retrospective Review	101 crush injury	47.5% Male, 52.5% Female	Crush injuries occur when a body part is subjected to a high force or pressure. Crusy syndrome is a result of muscular compression, myocytes are damaged, followed by the process of rhabdomyolysis, and systemic organ dysfunction	NM	22.5 h (AKI group), 12 h (non-AKI group)
Görmeli Kurt et al. (2024) [62]	Turkey/Kahramanmaraş Earthquake	Retrospective observational	377 CS	51.7% Male, 48.3% Female	CS, often occurring when pressure is released from a crushed limb, are severe conditions linked with rhabdomyolysis	the creatine kinase (CK) value was increased 3-fold and there was concomitant end-organ damage	24.92 ± 1.62 h, 9 h (AKI group), 10 h (non-AKI group), 9 h (dialysis group), 12 h (non-dialysis group)
Gunal et al. (2004) [63]	Turkey/Bingol Earthquake	Retrospective observational	16	75% Male, 25% Female	Patients with crush injury and systemic manifestations	serum levels of CK more than 1000	10.3 ± 7 h
Ilkay Guner et al. (2013) [64]	Turkey/Van Earthquake	Retrospective observational, descriptive analysis	46 CS	57% Male, 43% Female	Crush injury: compression of extremities and other body parts. CS: a localised crush injury with systemic manifestations	muscle swelling and/or neurological disturbances	NM
Gur et al. (2024) [65]	Turkey/Kahramanmaraş Earthquake	Retrospective observational	299	48.5% Male, 51.5% Female	Crush injuries occur when striated muscle cells are destroyed due to compression of muscle-rich areas in the body such as the extremities or trunk, CS is the disruption of cellular integrity and metabolic changes caused by the compression of skeletal muscles.	creatinine kinase (CK) level greater than 5 times the upper limit of normal	
He et al. (2011) [66]	China/Wenchuan Earthquake	cross-sectional	1,827 victims, 149 CS	51% Male, 49% Female	the presence of swollen limbs and history of limb compression	involvement of muscle mass, prolonged compression (usually 4–6 h, but possibly 1 h), compromised local circulation	7.7 h
Hosseini et al. (2009) [67]	Iran /Bam Earthquake	Retrospective observational	2962 (611 crushed: 200 CS, 411 crush injury)	60% Male, 40% Female	Crush injury with AKI or systemic complications	CPK > 1000 IU/L + systemic involvement	4.8 h (crush injury), 6.6 h ADDIN EN.CITE (1)
Huang et al. (2002) [68]	Taiwan/Chi-chi Earthquake		95	63% Male, 37% Female		hypovolemic shock, hyperkalemia, and acute renal failure a serum creatine kinase (CK) elevation to more than 1,000 U/L, within 2 weeks following the earthquake	7.1 ± 5.4 h, 9.9 ± 6.0 h (fasciotomy group), 5.2 ± 4.0 (non-fasciotomy group)
Najafi et al. (2010) [69]	Iran/Bam Earthquake		638	58.1% Male, 41.9% Female	moderate rhabdomyolysis: patients with 1000 ≤ creatine phosphokinase (CPK)< 15,000 IU/L (mean, 7000), and severe ones as CPK of at least 15,000.	moderate rhabdomyolysis: patients with 1000 ≤ creatine phosphokinase (CPK)< 15,000 IU/L (mean, 7000), and severe ones as CPK of at least 15,000.	6.3 ± 3.1 h (AKI group), 2.4 ± 1.6 h (non-AKI group)
Iskit et al. (2001) [70]	Turkey/Marmara Earthquake	Retrospective observational	33 (18 non-crush injury, 15 crush injury), 10 CS	51.5% Male, 48.5% Female	crush-injured patients with myoglobinuria or ARF were considered as having CS		30.04 ± 6.48 h, 35.44 ± 13.34 h (CS group), 34.38 ± 9.77 h (crush injury group), 27 ± 7.06 h (non-crush injury group)
Kantarci et al. (2002) [71]	Turkey/Marmara Earthquake	Retrospective analysis	476 (87 AKI)	60% Male, 40% Female	crush injury results in a characteristic syndrome with rhabdomyolysis inducing myoglobinuric acute renal failure, named CS. Compartment syndrome, defined as lack of perfusion of a limb and the disappearance of distal pulses.	NM	9.4 ± 6.9 h (dialysis group), 19.1 ± 22.5 h (non-dialysis group)
Kaya et al. (2024) [72]	Turkey/Kahramanmaraş Earthquake	Retrospective	82 crush-related AKI	54.9% Male, 45.1% Female	CS is a systemic manifestation of traumatic muscle injury	NM	NM
Kazancioglu et al. (2001) [73]	Turkey/Marmara Earthquake	Retrospective observational	60 CS	50% Male, 50% Female	CS is characterized by rhabdomyolysis, hypovolemic shock, hyperkalemia and acute renal failure occurring after any extensive muscle injury	urine output <400 ml/day and/or BUN >40 mg/dl, serum creatinine >2.0 mg/dl, uric acid >8.0 mg/dl, potassium >6.0 mEq/L, phosphorous >8.0 mg/dl and/or serum total calcium <8.0 mg/dl)	12.3 ± 15.1 h ADDIN EN.CITE (1), 8.6 ± 3.7 h (dialysis group), 20.4 ± 24.6 h (non-dialysis group)
Hafeez Kiani et al. (2015) [74]	Pakistan/Pakistan Earthquake	Retrospective observational	148 (15 within 24 h, 133 after 24 h) (18 crush injury,7 CS)	40% Male, 60% Female	NM	NM	NM
Köroğlu et al. (2024) ADDIN EN.CITE [40]	Turkey/Kahramanmaraş Earthquake	Retrospective	33 CS	51.5% Male, 48.5% Female	patients trapped under collapsed buildings based on a significant number of skeletal muscle injuries caused by being crushed by heavy materials, with or without swelling	serum CK levels exceeding 1000 U/L, neurological deficits, which may include sensory or motor changes at the injury site, the presence of urine discoloration	12 h (AKI group), 2 h (non-AKI group)
Koyuncu et al. (2023) [25]	Turkey/Kahramanmaraş Earthquake		237 CS	53.2% Male, 46.8% Female	systemic manifestations that are induced by crush injury are referred to as CS	NM	8 h
Kundakci et al. (2024) [33]	Turkey/Kahramanmaraş Earthquake	Retrospective observational	233 CS	47.6% Male, 52.4% Female	CS is the systemic manifestation of rhabdomyolysis resulting from pressure or crushing	NM	41.89 ± 29.75 h
Kurt et al. (2003) [75]	Turkey/Marmara and Düzce Earthquakes		75 crush injury (43 compartment syndrome)	54.5% Male, 45.5% Female	CS is a general manifestation of crush injury with presence of myoglobinuria with or without acute renal failure		14 ± 10 h in Marmara, 6 ± 4 h in Düzce
Li et al.(2021) [76]	Taiwan/Taiwan Earthquake	Retrospective observational	87 (31 rescued: WJ, 56 injured control)	48% Male, 52% Female	NM	patients were diag- nosed with rhabdomyolysis when the blood creatine kinase (CK) was more than 5 times the upper limit of the normal value	NM
Li et al. (2010) [77]	China/Wenchuan Earthquake		1030 (1012 victim, 18 healthy)	50% Male, 50% Female	the presence of swollen limbs and history of limb compression	Greaves et al. [78] & Gonzalez et al. [11]	NM
Group A (victims without crush syndrom(CS) and AKI)			904	50.3% Male, 49.7% Female		NM	NM
Group B (patients with CS and AKI who haven’t received renal replacement therapy (RRT)			57	45.6% Male, 54.4% Female		NM	NM
Group C (patients with CS and AKI receiving RRT)			25	52% Male, 48% Female		NM	NM
Group D (victims with AKI but without CS)			26	42.3% Male, 57.7% Female		NM	NM
Group E (18 healthy adult controls)			18	44.4% Male, 55.6% Female		NM	NM
Li et al. (2009) [34]	China/Wenchuan Earthquake	Retrospective observational	32 crush injury (17 CS)	65% Male, 35% Female	swelling and distension of limbs, dyscinesia, myoglobinuria, and hyperpotassemia, usually caused by prolonged pressing of body parts	over one hour pressing of the body parts; involvement of large amount of muscular tissue; development of pallor, clamminess, cold skin, pulselessness, or shock and the development of manifestations of acute renal failure	31 ± 12 h
Matsuoka et al. (2001) [79]	Japan/Hanshin Earthquake	Retrospective observational	42 CS	43% Male, 57% Female	hemodynamic and metabolic disturbances and acute renal failure following muscle injury due to prolonged compression of a limb	(1) compression of limb muscles; (2) swelling and neurologic disturbance of the affected area; and (3) presence of an abnormal urine finding, such as anuria, myoglobinuria, or hematuria	8.3 ± 4.5 h (fasciotomy group), 6.2 ± 2.0 h (non-fasciotomy group)
Moitinho de Almeida et al. (2019) [80]	Nepal/ Gorkha Earthquake	Retrospective descriptive	501 (21 crush injury)	48% Male, 52% Female	NM	NM	NM
Najafi et al. (2009)	Iran/Bam Earthquake		107 fasciotomy	60.2% Male, 38.8% Female	Crush injury with AKI or systemic complications, Pulselessness, Paresthesia, Paresis, Pallor, Pain	NM	5.06 ± 0.7 h
Nepali et al. (2017) [81]	Nepal/ Gorkha Earthquake		572	NM	rhabdomyolysis is a syndrome characterized by muscle necrosis and the release of intracellular muscle constituents, namely creatine phosphokinase, myoglobin, and various electrolytes into the circulation	NM	NM
Bonomini et al. (2011) [82]	Italy/L’Aquila Earthquake	NM	10	70% Male, 30% Female	NM	CS Patients Questionnaire by the Renal Disaster Relief Task Force	8 h
Safari et al. (2017) [83]	Iran/Bam Earthquake	Retrospective cross-sectional	135 CS	56.3% Male, 43.7% Female	traumatic rhabdomyolysis leading to serum creatinine over 1.66 mg/dl and CPK higher than 1000 IU/L in 2 measurements during hospitalization	serum creatinine over 1.66 mg/dl and CPK higher than 1000 IU/L	6.2 ± 3.4 h
Hatamizadeh et al. (2006) [84]	Iran/Bam Earthquake	NM	2086/554 (ARF and non-ARF patients)	53.2% Male, 46.8% Female	continuous and prolonged pressure on muscles	NM	6.2 ± 4.1 h (ARF group), 2.1 ± 3.9 h (non-ARF group), 6.1 ± 3.3 h (dialysis group), 6.7 ± 6.9 h (non-dialysis group)

**Table 2:** Laboratory parameters of affected individuals

Authors (Year)	Creatine kinase (CK)	Creatinine	BUN	Urinary output	Uric acid	Potassium	Phosphorus	Calcium	WBC
Adachi et al. (1998) [7]	> 1793 U/l (CS-AKI), > 1793 (Crush injury-non-AKI)	4.7 ± 2.2 mg/dl (CS-AKI), 0.8 ± 0.1 (Crush injury-non-AKI)	66 ± 23 mg/dl (CS-AKI), 11 ± 3 (Crush injury-non-AKI)	NM	NM	5.4 ± 1.0 mEq/l (CS-AKI), 3.9 ± 0.4 (Crush injury-non-AKI)	NM	NM	127 ± 38 (x10²/mm³) (CS-AKI), 89 ± 35 (x10²/mm³) (Crush injury-non-AKI)
Akbaba et al. (2023) [51]	17,113 IU/l (in dialysis group), 1112 (in non-dialysis group)	3.55 mg/dl (in dialysis group), 0.36 (in non-dialysis group)	NM	Reduced	NM	4.09 mEq/l (in dialysis group), 4.19 (dialysis not required)	NM	hypocalcemia	“ 14.3 (x10³/μL) (in dialysis group), 9.4 (x10³/μL) (in non-dialysis group)
Akgun et al. (2023) [52]	23868.50 IU/l (SSI group), 11812.50 (non-SSI group)	NM	NM	NM	NM	NM	NM	NM	NM
Aoki et al. (2006) [49]	NM	NM	NM	NM	NM	≥5 mmol/L (Hyperkalemia was a risk factor in secondary model)	NM	NM	≥18,000/mm³ (Risk factor in secondary model)
Atef et al. (1994) [26]	2975 U/l (AKI group), 1200 U/l (non-AKI group)	Elevated in ARF patients	Elevated in ARF patients	Measured; oligoanuria/anuria transferred to renal ward	NM	elevated in ARF patients	elevated in ARF patients	NM	NM
Aydin et al. (2024) [37]	7949 IU/L	2 mg/dL	50 mg/dL	1800 ml/day	10.3 mg/dL	5.2 ± 1.0 mEq/L	6.7 ± 2.9 mg/dL	8.9 mg/dL	18.9 × 10³/μL
Bakkaloglu et al. (2024) [55]	18,507 IU/l, 54 725 (AKI group), 7339.5 (non-AKI group), 85,390 (in dialysis group), 6253 (in non-dialysis group)	0.61 mg/dl, 1.9 (AKI group), 0.42 (non-AKI group), 2.89 (in dialysis group), 0.49 (in non-dialysis group)	NM	NM	5.64 mg/dl, 10.4 (AKI group), 3.7 (non-AKI group), 10.76 (in dialysis group), 4.16 (in non-dialysis group)	4.6 mmol/l, 5.4 (AKI group), 4.2 (non-AKI group), 6.1 (in dialysis group), 4.25 (in non-dialysis group)	NM	NM	NM
Demirkiran et al. (2002) [8]	NM	peaked in 12 patients (max 6.04 ± 4.22 mg/dl)	NM	Oliguria occurred in 8 patients	NM	Hyperkalaemia in 6 patients (max 5.35 ± 1.23 mEq/l)	NM	NM	Performed daily
Derici et al. (2002) [38]	37,472 ± 31,096.2 IU/l ADDIN EN.CITE (1), 855 ± 650 (non-CS)	406.6 ± 194.5 μmol/l ADDIN EN.CITE (1), 79.6 ± 8.8 (non-CS)	18.5 ± 7.4 mmol/l ADDIN EN.CITE (1), 5 ± 1.7 (non-CS)	5 patients were non-oliguric on admission.	NM	6.3 ± 1.5 mmol/l ADDIN EN.CITE (1), 4 ± 0.9 (non-CS)	2.16 ± 0.55 mmol/l ADDIN EN.CITE (1), 1 ± 0.36 (non-CS)	1.75 ± 0.33 mmol/l ADDIN EN.CITE (1), 2.2 ± 0.25 (non-CS)	NM
Döven S et al. (2024) [28]	significantly elevated in CS patients compared to non-CS	significantly elevated in AKI patients.	Significantly higher in CS patients	Oliguria present in 3.9% of all patients	Significantly higher in CS patients	Significantly higher in CS patients	Significantly higher in CS patients	Significantly lower in CS patients	Significantly higher in CS patients
Dönmez et al. (2001) [39]	18,099 ± 60,105 IU/l	1.7 ± 0.4 mg/dl	59.2 ± 10.9 mg/dl	1168 ± 203 ml/day	5.3 ± 0.4 mg/dl	5.6 ± 0.3 mEq/l	5.2 ± 0.3 mg/dl	8.2 ± 0.2 mg/dl	14,940 ± 1286/mm³
Ensari e al. (2002) [59]	39,507 ± 7,881 IU/l (in dialysis group), 17,810 ± 2,769 (in non-dialysis group)	4.74 ± 0.55 mg/dl (in dialysis group), 2.87 ± 0.14 (in non-dialysis group)	86.3 ± 5.23 mg/dl (in dialysis group), 62.8 ± 3.98 (in non-dialysis group)	NM	NM	5.94 ± 0.46 mmol/l (in dialysis group), 5.33 ± 0.21 (in non-dialysis group)	5.6±0.58 mg/dl (in dialysis group), 4.48±0.34 (in non-dialysis group)	NM	NM
Erek et al. (2002) [45]	23,443 ± 45,778 IU/ml	4.48 ± 2.95 mg/dl	60.6 ± 36.8 mg/dl	748 ± 1131 ml/day	6.5 ± 2.6 mg/dl	5.3 ± 1.3 mEq/l	5.2 ± 2 mg/dl	7.8 ± 1 mg/dl	14,571 ± 6535/mm³
Ersoy et al. (2002) ADDIN EN.CITE [46]	peak CPK: 6,887 ± 2,702 IU/l (survivors), 9,844 ± 2,035 (non-survivors)	3.7 ± 0.2 mg/dl, peak cr: 6.5 ± 0.3 (survivors), 4.9 ± 0.3 (non-survivors)	50.8 ± 2.9 mg/dl	63.3% oliguria	NM	5.7 ± 0.1 mEq/l, peak potassium: 6.0 ± 0.1 (survivors), 6.6 ± 0.3 (non-survivors)	NM	NM	NM
Hu et al. (2010) [61]	36,069 IU/l (AKI group), 4378 (non-AKI group)	NM	NM	NM	NM	NM	NM	NM	14.72(×10^9^/L) (AKI group), 10(×10^9/L) (non-AKI group)
Görmeli Kurt et al. (2024) [62]	20,738.17 ± 2,214.01 IU/l, 12,938 (AKI group), 3,758 (non-AKI group), 3,895 (dialysis group), 8,941 (non-dialysis group)	1.5 ± 0.1 mg/dl, 3.88 (AKI group), 0.53 (non-AKI group), 0.55 (dialysis group), 3.63 (non-dialysis group)	59.53 ± 3.56 mg/dl, 120 (AKI group), 27.5 (non-AKI group), 28 (dialysis group), 112 (non-dialysis group)	NM	NM	4.43 ± 0.04 mEq/l, 4.9 (AKI group), 4.1 (non-AKI group), 4.1 (dialysis group), 4.9 (non-dialysis group)	4.29 ± 0.13 mg/dl, 6.1 (AKI group), 3.3 (non-AKI group), 3.3 (dialysis group), 6 (non-dialysis group)	7.13 ± 0.04 mg/dl, 7.6 (AKI group), 8.4 (non-AKI group), 8.4 (dialysis group), 7.6 (non-dialysis group)	13.73 ± 0.54 (×10^9^/L), 14.7 (×10^9^/L) (AKI group), 10.84 (×10^9^/L) (non-AKI group), 10.7 (×10^9^/L) (dialysis group), 14.7 (×10^9^/L) (non-dialysis group)
Gunal et al. (2004) [63]	803 ± 726 IU/l	1.2 ± 0.5 mg/dl	NM	NM	8.3 ± 2.7 mg/dl	4.8 ± 1.1 mg/dl	6.0 ± 1.2 mg/dl	6.9 ± 1.1 mg/dl	19,656 ± 9236/mm³
Ilkay Guner et al. (2013) [64]	NM	NM	NM	NM	NM	6.32 ± 0.5 mmol/dl	NM	NM	NM
Gur et al. (2024) [65]	38289.46 ± 48470.161 IU/l	NM	NM	NM	NM	NM	NM	NM	NM
He et al. (2011) [66]	5,260 IU/l (in CS group), 133 (in non-CS group)	151.3 ± 1367 μmol/l (in CS group), 82.1 ± 64.1 (in non-CS group)	11.34 ± 9.65 mmol/l (in CS group),6.87 ± 5.88 (in non-CS group)	NM	308 ± 212 μmol/l (in CS group), 237 ± 117 (in non-CS group)	4.35 ± 1.04 mmol/l (in CS group), 3.91 ± 0.49 (in non-CS group)	1.27 ± 0.55 mmol/l (in CS group), 1.13 ± 0.36 (in non-CS group)	1.99 ± 0.35 mmol/l (in CS group), 2.21 ± 0.23 (in non-CS group)	9.73 ± 4.51 (×10^9^/L) (in CS group), 7.81 ± 3.70 (×10^9/L) (in non-CS group)
Hosseini et al. (2009) [67]	7000 IU/L (crush injury group), 25,561 IU/L (CS group)	1.6 mg/dL (crush injury group), 4.5 mg/dL (CS group)	36.6 mg/dL (crush injury group), 88.8 mg/dL (CS group)	NM	4.8 mg/dL (crush injury group), 8.7 mg/dL (CS group)	4.6 mEq/L (crush injury group), 6.09 mEq/L (CS group)	3.6 mg/dL (crush injury group), 5.6 mg/dL (CS group)	7.2 mg/dL (crush injury group), 6.05 mg/dL (CS group)	NM
Huang et al. (2002) [68]	48,158 ± 66,930 IU/l, 70,483 ± 78,543 (fasciotomy group), 35,135 ± 55,802 (non-fasciotomy group)	475 ± 330 μmol/l (fasciotomy group), 252 ± 286 (non-fasciotomy group)	NM	NM	NM	NM	NM	NM	NM
Najafi et al. (2010) [69]	26,706 ± 29,146 IU/l (AKI group), 4084 ± 6004 (non-AKI group)	NM	NM	NM	NM	NM	NM	NM	NM
Iskit et al. (2001) [70]	6,040 ± 4,158 U/L,6040 ± 4158.4 (crush injury-AKI group), 5912.± 6 4445.4 (crush injury-non-AKI group)	2.5 ± 1.6 mg/dl (crush injury-AKI group), 0.64 ± 0.15 (crush injury-non-AKI group)	73.22 ± 43.56 mg/dl (crush injury-AKI group), 21 ± 13.4 (crush injury-non-AKI group),	NM	NM	5.33 ± 1.34 mEq/l (crush injury-AKI group), 4.12 ± 0.7 (crush injury-non-AKI group)	NM	hypocalcemia in 6 cases of severe crush injury	NM
Kantarci et al. (2002) [71]	4,977 ± 8,264 IU/l (dialysis group), 1,341 ± 1,843 (non-dialysis group)	5.1 ± 3.8 mg/dl (dialysis group), 2.2 ± 1.7 (non-dialysis group)	69 ± 55 mg/dl (dialysis group), 44 ± 28 (non-dialysis group)	453 ± 489 ml/day (dialysis group), 1,484 ± 1,382 (non-dialysis group)	NM	5.5 ± 1.3 mEq/l (dialysis group), 4.6 ± 0.8 (non-dialysis group)	NM	7.5 ± 1 mg/dl (dialysis group), 8.3 ± 0.7 (non-dialysis group)	16,488 ± 9,156 /μL (dialysis group), 13,620 ± 5,135 (non-dialysis group)
Kaya et al. (2024) [72]	46,992 ± 5037IU/l	3.44 ± 2.07 mg/dl	67.1 ± 38.2 mg/dl	NM	9.9 ± 3.9 mg/dl	5.8 ± 1.2mmol/l	6.2 ± 2.6 mg/dl	7.2 ± 1.0 mg/dl	19.8 ± 9.7 (×10^3^/μL)
Kazancioglu et al. (2001) [73]	18453.1 ± 24527.2 IU/L, 24,333 ± 27,793 (dialysis group), 6693.2 ± 7239.1 (non-dialysis group)	4.4 ± 3.2 mg/dl	NM	1416.2 ± 2099.3 ml/day,823.6 ± 1839.8 (dialysis group), 2749.4 ± 2034.4 (non-dialysis group)	4.9 ± 3.2 mg/dl, 5.9 ± 3.4 (dialysis group), 2.7 ± 1.2 (non-dialysis group)	4.9 ± 1.7 mEq/L, 5.2 ± 1.9 (dialysis group), 4.4 ± 0.9 (non-dialysis group)	5.1 ± 1.9 mg/dl, 5.6 ± 1.9 (dialysis group), 3.9 ± 1.2 (non-dialysis group)	6.6 ± 1.9 mg/dl, 6.5 ± 2.2 (dialysis group), 6.9 ± 1.4 (non-dialysis group)	NM
Köroğlu et al. (2024) ADDIN EN.CITE [40]	initial: 35,000 IU/l (AKI group), 1707 h (non-AKI group)	initial: 2 mg/dL (AKI group), 0.9 (non-AKI group)	initial: 30.19 ± 14.12 mg/dL (AKI group), 14.24 ± 4.33 (non-AKI group)	NM	initial: 9 mg/dl (AKI group), 5.1 (non-AKI group)	initial: 5.62 mEq/L (AKI group), 4.18 (non-AKI group)	NM	initial: 8.63 ± 1.22 mg/dL (AKI group), 8.81 ± 0.94 (non-AKI group)	NM
Koyuncu et al. (2023) [25]	8628 IU/l ADDIN EN.CITE (1)	0.8 mg/dl ADDIN EN.CITE (1)	20 mg/dl ADDIN EN.CITE (1)	NM	4.4 mg/dl ADDIN EN.CITE (1)	4.5 ± 0.9 mmol/l ADDIN EN.CITE (1)	3.3 mg/dl ADDIN EN.CITE (1)	7.5 ± 1.1 mg/dl ADDIN EN.CITE (1)	12,000 (×10^9^/L) ADDIN EN.CITE (1)
Kundakci et al. (2024) [33]	69.817.69 ± 134.812.04 U/L	NM	NM	NM	NM	NM	NM	NM	NM
Li et al. (2010) [77]									
Group A (victims without crush syndrom(CS) and AKI)	166	80.4 ± 63.6 μmol/l	6.05 ± 3.83 mmol/L	NM	233.9 ± 113.5 μmol/l	3.93 ± 0. 51 mmol/L	1.13 ± 0.42 mmol/L	2.20 ± 0.22 mmol/L	8.01 ± 3.81 (×10^9^/L)
Group B (patients with CS and AKI who haven’t received renal replacement therapy (RRT)	216	163.2 ± 73.6μmol/l	6.05 ± 3.83 mmol/L	NM	348.0 ± 234.5 μmol/l	4.12 ± 0.80 mmol/L	1.31 ± 0.56 mmol/L	2.08 ± 0.32 mmol/L	9.25 ± 4.36 (×10^9^/L)
Group C (patients with CS and AKI receiving RRT)	18,636	419.1 ± 141.5 μmol/l	22.90 ± 10.62 mmol/L	NM	486.1 ± 230.2 μmol/l	5.80 ± 1.27 mmol/L	1.74 ± 0.76 mmol/L	1.71 ± 0.25 mmol/L	11.12 ± 6.00 (×10^9^/L)
Group D (victims with AKI but without CS)	NM	157.7 ± 57.9 μmol/l	11.37 ± 5.46 mmol/L	NM	393.1 ± 224.3 μmol/l	3.79 ± 0. 59 mmol/L	1.33 ± 0.62 mmol/L	2.10 ± 0.38 mmol/L	NM
Group E (18 healthy adult controls)	NM	NM	NM	NM	NM	NM	NM	NM	NM
Li et al. (2009) [34]	4697 ± 359 IU/l	794 ± 85 umol/L	32.6 ± 12.8 mmol/L	NM	NM	5.4 ± 2.4 mmol/l	NM	NM	21,562 ± 8765 cells/μL
Matsuoka et al. (2001) [79]	101,000 ± 72,000 IU/l (fasciotomy group), 71,000 ± 55,000 (non-fasciotomy group)	NM	NM	NM	NM	NM	NM	NM	NM
Najafi et al. (2009)	19,416 ± 2975 IU/l (fasciotomy group), 14,184 ± 1692 (non-fasciotomy group)	Inappropriate data	Inappropriate data	Inappropriate data	Inappropriate data	Inappropriate data	Inappropriate data	Inappropriate data	Inappropriate data
Nepali et al. (2017) [81]	18144.39 ± 18589.76 IU/l	495.26 ± 319.92 μmol/l	NM	NM	NM	5.09 ± 1.31 mEq/l	NM	NM	NM
Bonomini et al. (2011) [82]	67,577 IU/l	2.61 mg/dl	49.2 mg/dl	930 ml/day	8.9 mg/dl	5.86 mEq/l	NM	7.44 mg/dl	17.97 (x10³/μL)
Safari et al. (2017) [83]	17.4 ± 24.7 IU/l	4.73 ± 2.3 mg/dl	104.0 ± 59.0 mg/dl	897.0 ± 923.0 ml/day	8.5 ± 2.8 mg/dl	5.6 ± 1.3 mEq/l	NM	NM	NM
Hatamizadeh et al. (2006) [84]	4,373.1 ± 10,005.7 U/L, 15,277.1 ± 19,550.0 IU/l (ARF group), 3,976.4 ± 7,670.5 (non-ARF group), 15,583.4 ± 19,564.7 (dialysis group), 14,968.8 ± 22,362.1 (non-dialysis group)	1.2 ± 1.4 mg/dL	NM	NM	NM	4.4 ± 0.8 mEq/L, 5.6 ± 1.2 mEq/L (ARF group), 4.3 ± 0.5 (non-ARF group), 5.7 ± 1.2 (dialysis group), 5.2 ± 0.9 (non-dialysis group)	NM	NM	NM

**Table 3:** Reported outcomes of affected individuals of the included studies

Authors (Year)	Sequelae of crush injury	Treatment Provided	Renal Complications / AKI	Dialysis Required	Mortality Rate
Adachi et al. (1998) [7]	AKI (15 out of 15 CS), DIC (3 out of 15 CS)	(CS-AKI): Continuous arterio-venous hemofiltration	15 out of 15 CS patients	46.7% (7 out of 15 CS)	20% (3 out of 15 in CS)
Akbaba et al. (2023) [51]	AKI 76% (19 out of 25 CS), infectious complications (25 out of ??), thromboembolic complications (5 out of ??), psychological complications (26 out of ??), upper respiratory tract infections 37.9% (69 out of 180),	IV fluids, hemodialysis, plasma exchange, blood transfusions, fasciotomy, amputation, alkalinized solutions, antibiotics and tetanus prophylaxis	76% (19 out of 25 CS)	56% (14 out of 25 CS)	NM
Akgun et al. (2023) [52]	Surgical Site Infections (SSI) 50% (58 out of 116), AKI 62.1% of SSI group (36 out of 58), 31.0% of non-SSI group (18 out of 58)	fasciotomy, prophylactic antibiotics, wound care, hyperbaric oxygen therapy	62.1% of SSI group (36 out of 58), 31.0% of non-SSI group (18 out of 58)	NM	NM
Akkoç et al. (2024) [53]	compartment syndrome 23.7%, multiple organ failure 2.6% (1 out of 38), upper extremity injury (38 out of 38)	multiple debridement, skin grafting, fasciotomy, amputation, vacuum‑assisted closure, stump revision, tendon/bone repair, wound care, fluid replacement, antibioc therapy, nutrition, psychotherapy, physiotherapy	NM	NM	2.6% (1 out of 38)
Aoki et al. (2006) [49]	AKI 55.4% (191 out of 345), extremities injuries	IV fluids	55.4% (191 out of 345)	37.7% (130 out of 345)	13.9% (48 out of 345)
Asfuroğlu et al. (2023) [54]	fractures (132 out of 204), soft tissue damage (9 out of 204)	surgical procedures (fracture fixation, debridement, amputation, fasciotomy) and non-surgical treatments, implants	NM	NM	1.47% (3 out of 204)
Atef et al. (1994) [26]	AKI 6% (30 out of 495), peripheral nerve damage (55% in AKI patients), multiple injuries (100% in AKI patients), compartment syndrome	If the patient was dehydrated, large boluses of fluid were given based on age, weight and severity of dehydration. If the patient was well hydrated, they were given furosemide 40 mg, and if ineffective, 100 mg IV, fasciotomy	6% (30 out of 495)	37.6% (186 out of 495)	7.5% (37 out of 495)
Aydin et al. (2024) [37]	AKI 88.7% (55 out of 62), compartment syndrome 38.7% (24 out of 62), infection 87.1% (54 out of 62), sepsis 87.1% (54 out of 62), septic shock 35.5% (22 out of 62), DVT 1.6% (1 out of 62), fractures 61.3% (38 out of 62), DIC, pneumothorax 24.2% (15 out of 62), hemothorax 11.3% (7 out of 62), pneumomediastinum 16.1% (10 out of 62), trauma-induced				
Bakkaloglu et al. (2024) [55]	AKI 34.8% (314 out of 903), multiorgan dysfunction (14 out of 903), sepsis (1 out of 903), pneumomediastinum (4 out of 903), pulmonary hemorrhage (1 out of 903), cerebral salt wasting/brain edema (1 out of 903)	IV fluids (types and volumes specified), surgeries, fasciotomy, amputation, dialysis, ventilator, inotropic support	34.8% (314 out of 903)	20.9% (189 out of 903)	2.4% (22 out of 903)
Çağıran et al. (2022) [56]	CS, AKI, compartment syndrome, drop foot, shoulder dislocation, droopy eyelid, dermatitis	osteosynthesis, fixation, fasciotomy, amputation, debridement, splinting	8.55% (13 out of 152)	1.32% (2 out of 152)	25.7% (39 out of 152)
Xiaolei et al. (2010) [27]	AKI 81% (47 out of 58), wound infection 55.2%, sepsis 44.8% (26 out of 58), multiple organ dysfunction, extremities injuries 93.1%,	fasciotomy, amputation, renal replacement therapy (primarily hemodialysis, CRRT/CVVH), antibiotics in infections(cephalosporins, carbapenems, quinolones)	81% (47 out of 58)	81% (47 out of 58)	6.9% (4 out of 58)
Del Papa et al. (2019) [57]	fractures (46.8%), crushing injuries (2.9%), internal injuries (14.6%)	Not detailed	NM	NM	1.8% (3 out of 171)
Demir et al. (2024) [42]	Peripheral nerve damage (31 out of 36), CS (61.1%), compartment syndrome (19.4%)	Not detailed	NM	NM	NM
Demirkiran et al. (2002) [8]	DIC, sepsis (2 out of 18), AKI (13 out of 18), multiple organ failure, ARDS (4 out of 18), pneumothorax (1 out of 18), pericardial effusion (1 out of 18), pulmonary embolism (1 out of 18), pleural effusion (1 out of 18)	fasciotomy, amputations, mechanical ventilation, haemodialysis, haemoperfusion, continuous haemofiltration	13 out of 18	13 out of 18	8 out of 18
Derici et al. (2002) [38]	AKI (17 out of 17), DIC (7 out of 17), pulmonary embolism (5 out of 17), pneumonia (8 out of 17), wound infection (12 out of 17)	hemodialysis, fasciotomy	17 CS	15 out of 17 CS	5 out of 17 (29.4%)
Döven S et al. (2024) [28]	AKI 23.7% (14 out of 59 CS), compartment syndrome 27.1% (16 out of 59 CS), soft tissue injuries 72.9% (43 out of 59 CS), pneumothorax 8.5% (5 out of 59 CS), Extremity fractures 28.8% (17 out of 59 CS)	IV fluid, hemodialysis, fasciotomy, amputation, hyperbaric oxygen, albumin, erythrocyte suspension, fresh-frozen plasma, intubation	2.6% (17 out of 649), 23.7% of (14 out of 59 CS)	(7 of 14 AKI patients with CS), 10 out of 649 (1.5%)	0%
Dönmez et al. (2001) [39]	AKI 35% (7 out of 20), wound infection (3 out of 20), sepsis (1 out of 20)	IV fluid, diuretics, alkaline therapy, fasciotomy, hemodialysis, albumin, plasma infusions, packed erythrocyte infusions	35% (7 out of 20)	4 out of 7 ARF patients	1 out of 20
Ellidokuz et al. (2005) [58]	injuries 2.2% (18 out of 812), extremities injury 56% (10 out of 18)	Not detailed	NM	NM	1.6% (13 out of 812)
Ensari e al. (2002) [59]	AKI (27 out of 27 CS), extremities injuries (27 out of 27 CS)	IV fluids, mannitol and diuretics, fasciotomy (17 out of 27), dialysis	27 CS patients	10 out of 27 CS	NM
Erek et al. (2002) [45]	AKI (639 out of 639), fractures 22.2% (142 out of 639), thoracic and abdominal trauma 17.2% (110 out of 639), infection (34.9%), sepsis (18.9%), DIC (6.9%), thromocytopenia (5.8%), ARDS (7.3%), pleural effusion (3.4%), CHF (2.8%), hypervolaemia (0.6%), hypovolaemia shock (0.46%), GI problems (3.56%), peripheral neuropathy (4.8%)	IV fluids, fasciotomy, dialysis, amputation	639 out of 639	74.6% (477 out of 639)	17.2% (in dialysis group), 9.3% (in non-dialysis group)
Ersoy et al. (2002) ADDIN EN.CITE [46]	AKI (60 out of 60), limb trauma (60 out of 60), multiple trauma (10 out of 60), wound infection, sepsis (27 out of 60), renal contusion (1 out of 60), intestinal injury (1 out of 60), urinary bladder rupture (2 out of 60), postpyloric ulcus perforation (1 out of 60), ARDS (1 out of 60), DIC, retroperitoneal hematoma (2 out of 60), multiple organ failure (14 out of 60)	renal replacement therapy, albumin, dextrose or hypotonic sodium chloride solutions, fasciotomy, amputation, fresh-frozen plasma, whole blood transfusions	60 out of 60 patients	60 out of 60 patients	35% (21 out of 60)
Fan et al. (2010) [60]	soft tissue injuries (48%), fractures (40%), shock, wound infection, hemorrhagic shock, multi organ dysfunction	amputation (14 out of 1038)	NM	NM	NM
Hu et al. (2010) [61]	AKI 41.5% (42 out of 101), infections (46.5%), sepsis (9%), pulmonary infection (15.8%), urinary infection (3%), shock (3%), ARDS (3%)	fasciotomy, amputation	41.5% (42 out of 101)	NM	5% (5 out of 101), 4 out of 42 AKI group
Görmeli Kurt et al. (2024) [62]	AKI, infections (46.5%), sepsis (9%), pulmonary infection (15.8%), urinary infection (3%), shock (3%), ARDS (3%)	aggressive volume replacement followed by forced diuretic therapy	23.1% (87 out of 377)	75.9% (286 out of 377)	5.3% (20 out of 377)
Gunal et al. (2004) [63]	AKI (16 out of 16), DIC (3 out of 16), Urinary tract infection (2 out of 16), peripheral neuropathy (14 out of 16), psychiatric problems (8 out of 16), ischemic encephalopathy (1 out of 16), fractures (5 out of 16), Hypertension (3 out of 16)	isotonic saline, mannitol alkaline fluid resuscitation, fasciotomy, cephalosporins and antianaerobic antibiotics, narcotic analgesics and proton pump inhibitors	16 out of 16	25% (4 out of 16)	0%
Ilkay Guner et al. (2013) [64]	AKI 60.9% (28 out of 46), compartment syndrome (16 out of 46), sepsis (7out of 46), wound infection (18 out of 46), pericardial effusion (3 out of 46), pleural effusion (2 out of 46), fracture (6 out of 46), haemothorax (3 out of 46), pulmonary embolism (1 out of 46)	fasciotomy, amputation	60.9% (28 out of 46)	34.7% (16 out of 46)	23.9% (11 out of 46)
Gur et al. (2024) [65]	AKI, severe extremities damage 54.5% (163 out of 299)	debridement, fasciotomy, amputation, hemodialysis	Yes	32.1% (96 out of 299)	11.1% (32 out of 299)
He et al. (2011) [66]	infection (19 out of 149), sepsis (2 out of 149), AKI 41.6% (62 out of 149 CS), 1.6% (27 out of 1678 non-CS), DIC (3 out of 149), ARDS (4 out of 149), congestive heart failure (6 out of 149), multiple organs dysfunction syndrome (5 out of 149), hypovolemic shock 10% (15 out of 149)	fasciotomy, amputation, ventilatory support, vasopressors, renal replacement therapy,	41.6% (62 out of 149 CS), 1.6% (27 out of 1678 non-CS)	22% (33 out of 149)	overall: 1%, 6.7% (10 out of 149), 0.8% (14 out of 1678)
Hosseini et al. (2009) [67]	AKI (200 out of 611), DIC (13 out of 611 crushed) (12 out of 200 CS), ARDS (18 out of 611 crushed) (17 out of 200 CS), extremities injuries, sepsis (21 out of 611 crushed) (20 out of 200 CS)	IV fluids, dialysis, ICU care	Yes (CS group)	(in severe cases)	(total: 48 out of 2962), 61% of deaths in crushed group (29 out of 48 death) (29 out of 611) (23 out of 200 CS)
Huang et al. (2002) [68]	fractures 45.1% (41 out of 95), motor/sensory neuropathy 34.1% (31 out of 95), AKI 46.3% (44 out of 95), compartment syndrome (35 out of 95), infection (24 out of 95)	fasciotomy, hemodialysis	46.3% (44 out of 95)	31.6% (30 out of 95)	8.4% (8 out of 95)
Najafi et al. (2010) [69]	AKI 21% (134 out of 638)	IV fluids, hemodialysis,	21% (134 out of 638)	17.2% (110 out of 638) (110 out of 134 AKI)	
Iskit et al. (2001) [70]	compartment syndrome (6 out of 15 crush injury), infection, multiorgan failure (1 out of 15 crush injury), AKI (10 out of 15 crush injury), Extremity fractures (3 out of 15 crush injury), soft tissue injury (7 out of 15 crush injury)	fasciotomy, conservative therapy, ventilatory support, fluid replacement, alkalinization, diuretic drugs	10 out of 15 crush injury	2 out of 15 crush injury	
Kantarci et al. (2002) [71]	AKI 18.2% (87 out of 476), limb injuries	fasciotomy, vigorous fluid administration, alkaline diuresis, mannitol, renal replacement therapy (hemodialysis), amputation, blood and fresh-frozen plasma transfusions	18.2% (87 out of 476)	68% (59 out of 87 AKI)	8 out of 87
Kaya et al. (2024) [72]	AKI (82 out of 82)	hemodialysis, IV fluids,	82 out of 82	82 out of 82	6.1% (5 out of 82)
Kazancioglu et al. (2001) [73]	AKI, fractures 22% (13 out of 60), multiple traumas (21 out of 60), extremity injuries 85% (51 out of 60)	fasciotomy, amputation, fluid resuscitation was initiated with mannitol-alkali solutions, alkali solutions and isotonic NaCl	Yes	40 out of 60	13 out of 60
Hafeez Kiani et al. (2015) [74]	musculoskeletal injuries (11 out of 15 within 24 h, 105 out of 133 after 24 h), wound infections (7 out of 15 within 24 h, 27 out of 133 after 24 h), pneumothorax 1.4% (2 out of 148), Hemothorax 0.7% (1 out of 148)	NM	NM	NM	0.7% (1 out of 148)
Köroğlu et al. (2024) ADDIN EN.CITE [40]	AKI 57.5% (19 out of 33), DIC 3% (1 out of 33), ARDS 6% (2 out of 33), sepsis 18.2% (6 out of 33), wound infection 18.2% (6 out of 33)	non-surgical treatment, fasciotomy, amputation, dialysis,	57.5% (19 out of 33)	36.4% (12 out of 33)	6% (2 out of 33)
Koyuncu et al. (2023) [25]	AKI 36.7% (87 out of 237), extremity traumas 35.8% (84 out of 237), abdominal trauma 8.4% (20 out of 148), thoracic trauma 13.6% (32 out of 148)	erythrocyte replacement, fresh-frozen plasma, hyperbaric oxygen therapy, fasciotomy, amputation, hemodialysis, IV fluids, mannitol, bicarbonate infusion	36.7% (87 out of 237)	29.9% (71 out of 237)	17.2%(41 out of 237)
Kundakci et al. (2024) [33]	extremities injuries, abdominal trauma 12.4% (29 out of 233), thoracic trauma 20.2% (47 out of 233)	fasciotomy (17.6%), amputation (30.9%),	Yes	56.7% (132 out of 233)	6.4% (15 out of 233)
Kurt et al. (2003) [75]	AKI, wound infection 37.3%, extremities fractures 24%, compartment syndrome (43 out of 75), sustained associated injuries 38.6% (29 out of 75)	fasciotomy(57%), IV fluids, furosemide, mannitol	Yes	35 out of 75	14.6% in both, 15.9% in Marmara and 8.3% in Düzce
Li et al.(2021) [76]	rhabdomyolysis (13 out of 87), AKI, fractures (28 out of 87), compartment syndrome (4 out of 31 WJ)	conservative treatment, fasciotomy,	5 out of 13 rhabdomyolysis	Yes (1 out of 5 AKI)	NM
Li et al. (2010) [77]	AKI (1012 out of 1030), malnutrition, inflammation	IV infusion, oral supplements			
Group C (patients with CS and AKI receiving RRT)	NM	NM	NM	NM	4% (1 out of 25)
Li et al. (2009) [34]	traumatic shock 56.25% (18 out of 32), AKI 34.38% (11 out of 32), acute heart failure (6 out of 32), stress ulcer 6.25% (2 out of 32), multiple organ dysfunction syndrome 12.5% (4 out of 32), severe infection 15.63% (5 out of 32), multiple injuries (27 out of 32), hemoglobinuria (23 out of 32), acute pulmonary edema 18.75% (6 out of 32), chest trauma (11 out of 32), retroperitoneal hematoma (3 out of 32)	hemodialysis, amputation 15.63%, antishock treatment, urine alkalization, fasciotomy, broad-spectrum antibiotics, tetanus antitoxin	34.38% (11 out of 32)	19 out of 32	18.75% (6 out of 32)
Matsuoka et al. (2001) [79]	AKI (34 out of 42), motor/sensory disturbance, muscle weakness, severe disability (12 out of 42)	debridement, fasciotomy,	34 out of 42	26 out of 42	none
Moitinho de Almeida et al. (2019) [80]	fractures 65.8% (288), limb injuries (246 out of 399)	surgery treatments, amputation,	NM	NM	7% (37 out of 501)
Najafi et al. (2009)	sepsis, DIC, ARDS,	fasciotomy, amputation, IV fluids,	65% (70 out of 107 fasciotomy patients)		
Nepali et al. (2017) [81]	AKI 4.02% (23 out of 572),sepsis 43.47% (10 out of 23 AKI), compartment syndrome 34.78% (8 out of 23 AKI), lower extremity injury (49 out of 572)	IV fluids, hemodialysis	4.02% (23 out of 572)	86.9% (20 out of 23 AKI)	3.32% (19 out of 572), 8.6% (2 out of 23 AKI)
Bonomini et al. (2011) [82]	AKI (10 out of 10), fractures (6 out of 10)	IV fluids, blood transfusions, dialysis, fres-frozen plasma, human albumin transfusion, fasciotomy, pleural drainage	10 out of 10	10 out of 10	0%
Safari et al. (2017) [83]	hypokalemia, hyperkalemia,	urine alkalinization, fluid resuscitation	NM	NM	NM
Hatamizadeh et al. (2006) [84]	sepsis (11.6% in ARF group, 0.5% in non-ARF group), ARDS (9.1% in ARF group, 1.4% in non-ARF group), DIC (7.3% in ARF group, 0.3% in non-ARF group), extremity injuries (720 out of 2086), AKI 30% (145 out of 484)	fasciotomy, amputation, IV fluids, dialysis,	30% (145 out of 484)	75.7% (106 out of 140)	5.05% totally, 12.7% (ARF group), 1.9% (non-ARF group)

### Synthesis of definitions and methodological considerations

Substantial clinical and methodological heterogeneity was observed, primarily stemming from inconsistent definitions of CS across studies. Definitions variably emphasized systemic manifestations of rhabdomyolysis, the presence of AKI following crush injury, or specific compartment syndrome symptoms. Notably, a substantial proportion of studies (approximately 40%) did not specify explicit diagnostic criteria for CS, recording this information as “not mentioned” (NM). This lack of diagnostic granularity represents a significant limitation: it precludes meaningful reclassification of cases, obscures the distinction between isolated crush injury and full-blown crush syndrome, and likely contributes to the inflation of between-study heterogeneity. Consequently, subgroup comparisons between crush injury and crush syndrome cohorts should be interpreted with caution, as misclassification bias cannot be excluded. Time under rubble, a critical prognostic factor, ranged widely from 2 to 31 h across cohorts.

Most studies (56%) were conducted in Turkey, focusing primarily on the Kahramanmaraş earthquake in 2023 and the Marmara earthquake in 1999. Six studies (12%) examine earthquakes in Iran, especially the Bam earthquake. China has been determined to have suffered a crush injury due to the Wenchuan earthquake in 6 studies.

Most studies have retrospective designs, which lead to low quality for numerous reasons, such as missing data. Other study designs, such as case-control, descriptive, and cross-sectional, include six studies, which are a small part of this systematic review.

The definition of CS varies across studies, resulting in substantial heterogeneity in this review. Some studies define it as a systemic consequence of muscle tissue damage (rhabdomyolysis) caused by pressure, some other articles mention the symptoms, including pallor, severe pain, paresthesia, pulselessness, and paralysis, and other studies indicate CS with AKI due to crush injury. The diagnostic criteria for CS are not clearly defined in most studies. Urine output < 400 ml/day and/or BUN > 40 mg/dl, serum creatinine > 2.0 mg/dl, uric acid > 8.0 mg/dl, potassium > 6.0 mEq/L, phosphorous > 8.0 mg/dl and/or serum total calcium < 8.0 mg/dl [18] are some of the diagnostic criteria that can assist doctors in diagnosing CS more quickly. Time spent under the rubble ranges from 2 to 31 h, and multiple complications are more likely to occur when the time spent under the rubble is longer.

Laboratory data such as Creatine kinase (CK), creatinine, BUN, urinary output, uric acid, potassium, phosphorus, calcium, and WBC are measured in most studies, especially CK, due to its importance in diagnosing CS. In almost all studies, the CK value is much higher than 1000 IU/L. Urinary output is measured in only six studies (12%), which is a little.

The sequelae of crush injuries are diverse, including mostly AKI in 78% of the articles (*n* = 39). ARDS was seen in 20%, DIC in 24%, compartment syndrome in 24%, extremity injury in 28%, and fractures in 26% of the studies. Other complications include pneumothorax, hemothorax, infections, limb injuries, multiple injuries, and multiple organ failure. Ten studies (20%) use acute renal failure (ARF) for the term of kidney injury, which can lead to misleading information and can cause confusion in analyzing data. For this, we used the AKI term to encompass all kidney damage across all studies, defined as a multifactorial syndrome with a high risk of short- and long-term complications and increased health care costs [19].

Early identification and management of CS are critical for achieving a significant reduction in complications and mortality [20]. The applied treatments in the studies are described in detail in Table 1. IV fluids were the most important and first-line treatment to maintain intravascular volume [21]. Hemodialysis, amputation, fasciotomy, ventilation support, plasma infusions, and packed erythrocyte infusions are other mentioned treatments. The gold standard treatment for CS is early fasciotomy of the affected limb [22].

Fasciotomy was commonly reported, particularly in patients with established compartment syndrome. While fasciotomy remains the standard of care for confirmed compartment syndrome [22], its prophylactic or liberal use in crush injury without objective compartment pressure elevation remains controversial [23], especially in disaster settings due to high infection risk, delayed wound healing, and resource constraints [24].

Hyperbaric oxygen was used in 3 studies as one of the treatments. Manitol and diuretics were applied to patients in 11 studies. Dialysis is the most common treatment for AKI in this review, and its rates are described in Table 1.

Across earthquake cohorts, nearly half of patients with AKI or crush-related renal injury required dialysis, overall mortality was approximately 8%, and a wide range of systemic and local complications were common (overall pooled sequelae = 25%). However, most pooled estimates were accompanied by high heterogeneity, reflecting variation in study populations (AKI vs. CS vs. mixed victims), case definitions, timing of assessment, and local disaster response practices. Formal tests did not show strong small-study bias for mortality, but heterogeneity limits the certainty of pooled proportions (see Supplementary CMA tables). Studies with sufficient data are mentioned in the tables.

### Methodological quality

Methodological quality, appraised using JBI checklists, was frequently limited by the inherent constraints of retrospective designs in disaster settings, including potential missing data and selection bias. These factors are considered in the interpretation of pooled estimates.

### Terminology clarification

Ten studies used the term *acute renal failure (ARF)*. For harmonization, all kidney injury outcomes were analyzed under the umbrella term *acute kidney injury (AKI)*, consistent with contemporary nomenclature and reflecting a multifactorial syndrome with significant short- and long-term consequences.

### Meta-analysis of primary outcomes

#### Dialysis requirement (acute dialysis / renal replacement therapy)

Thirty-eight study arms (n studies pooled = 38) contributed data on the need for dialysis after earthquake-related crush injuries. The overall pooled proportion requiring dialysis was 0.49 (95% CI 0.40–0.58), with high between-study heterogeneity (Q = 1214.26, df = 37, *p* < 0.001; I² = 96.95%). Subgroup analyses by clinical grouping showed higher dialysis use among studies reporting acute kidney injury (AKI) (pooled proportion 0.60; 95% CI 0.24–0.88; 3 studies) compared with studies of CS alone (0.46; 95% CI 0.20–0.75; 4 studies). Other studies had a pooled dialysis proportion of 0.49 (95% CI 0.38–0.59). Heterogeneity within subgroups was substantial for several categories (I² for CS = 78.8%) (Fig. 2a and 2b, and supplementary file 2).

**Fig. 2a:**
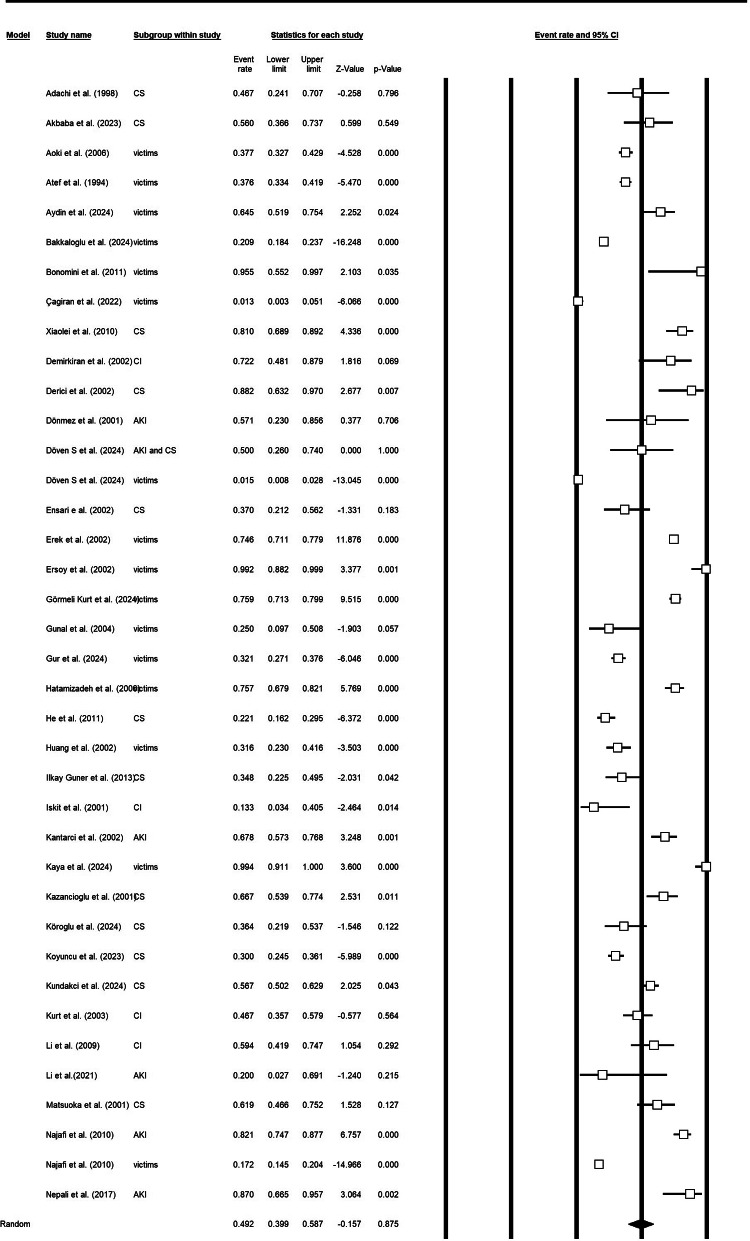
Dialysis requirement in survivors

**Fig. 2b:**
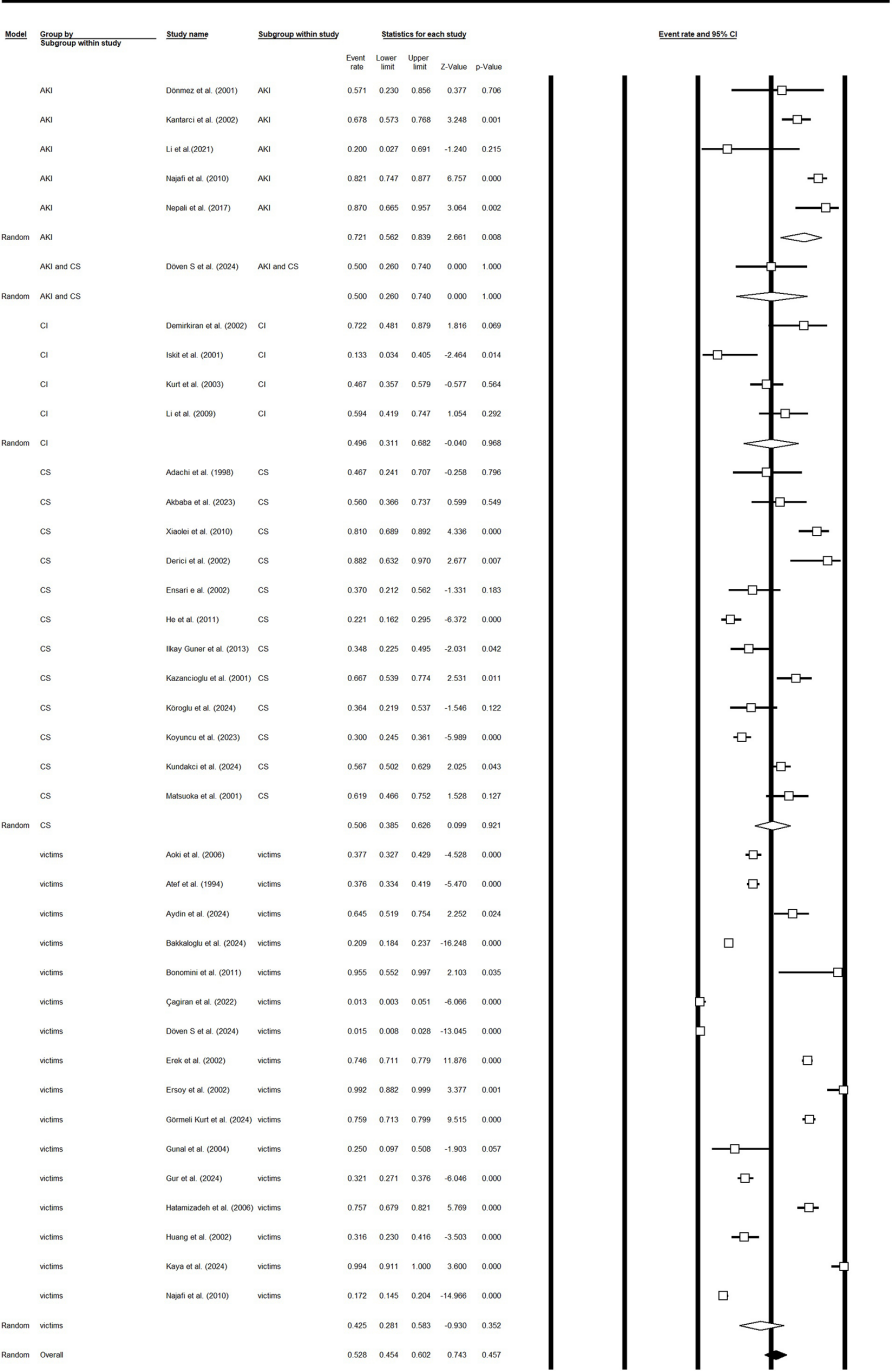
Dialysis requirement in survivors based on the CS or crush injury or only victims of earthquake

### Mortality

Thirty-nine study arms contributed mortality data. The pooled mortality proportion across all earthquake-related cohorts was 0.08 (95% CI 0.06–0.10) with substantial heterogeneity (Q = 545.76, df = 38, *p* < 0.001; I² = 93.0%). Subgroup estimates included: AKI cohorts 0.09 (95% CI 0.06–0.15; 3 studies; low heterogeneity I² ≈ 0%), crush-injury cohorts 0.17 (95% CI 0.04–0.50; 3 studies; I² = 88.4%), and crush-syndrome cohorts 0.12 (95% CI 0.07–0.18; 12 studies; I² = 84.8%). These results indicate that while overall mortality after earthquake-related crush injuries is modest at the pooled level (~ 8%), estimates vary considerably by clinical subgroup and study (Fig. 3a and 3b, and supplementary file 3).

**Fig. 3a:**
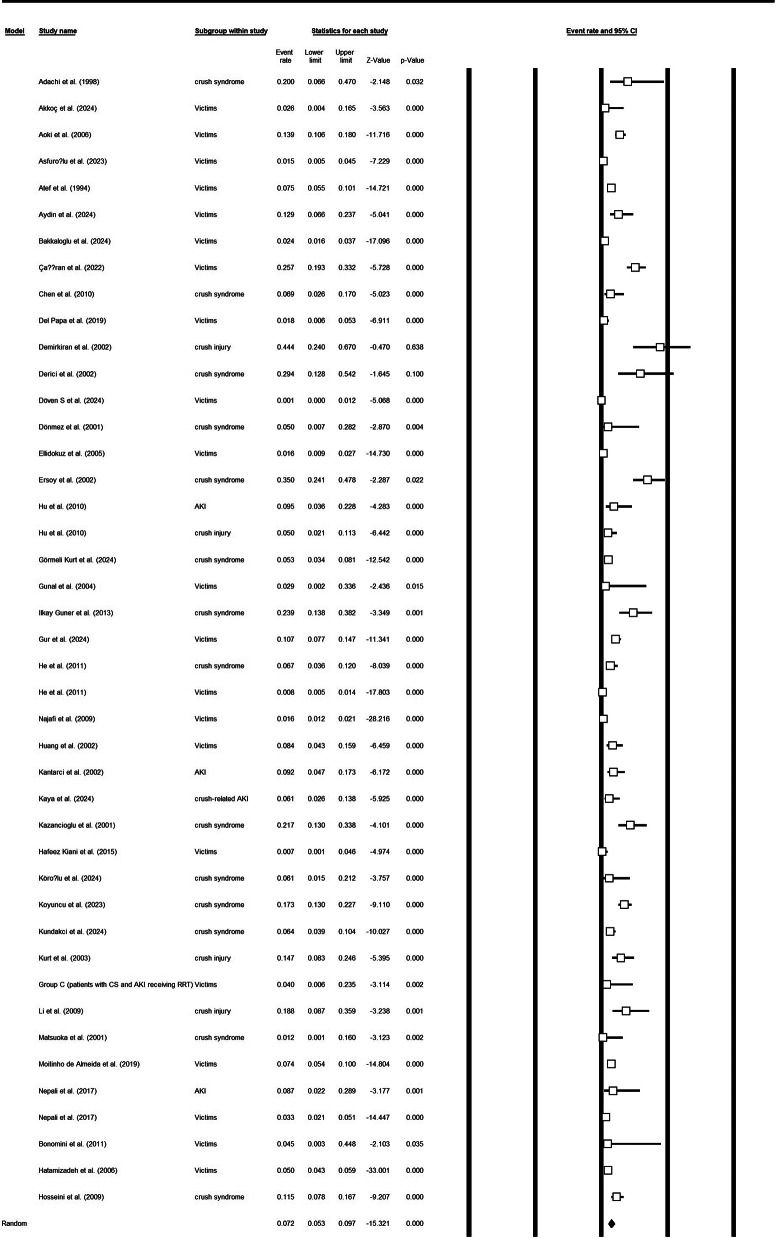
Total mortality rate in affected individuals

**Fig. 3b:**
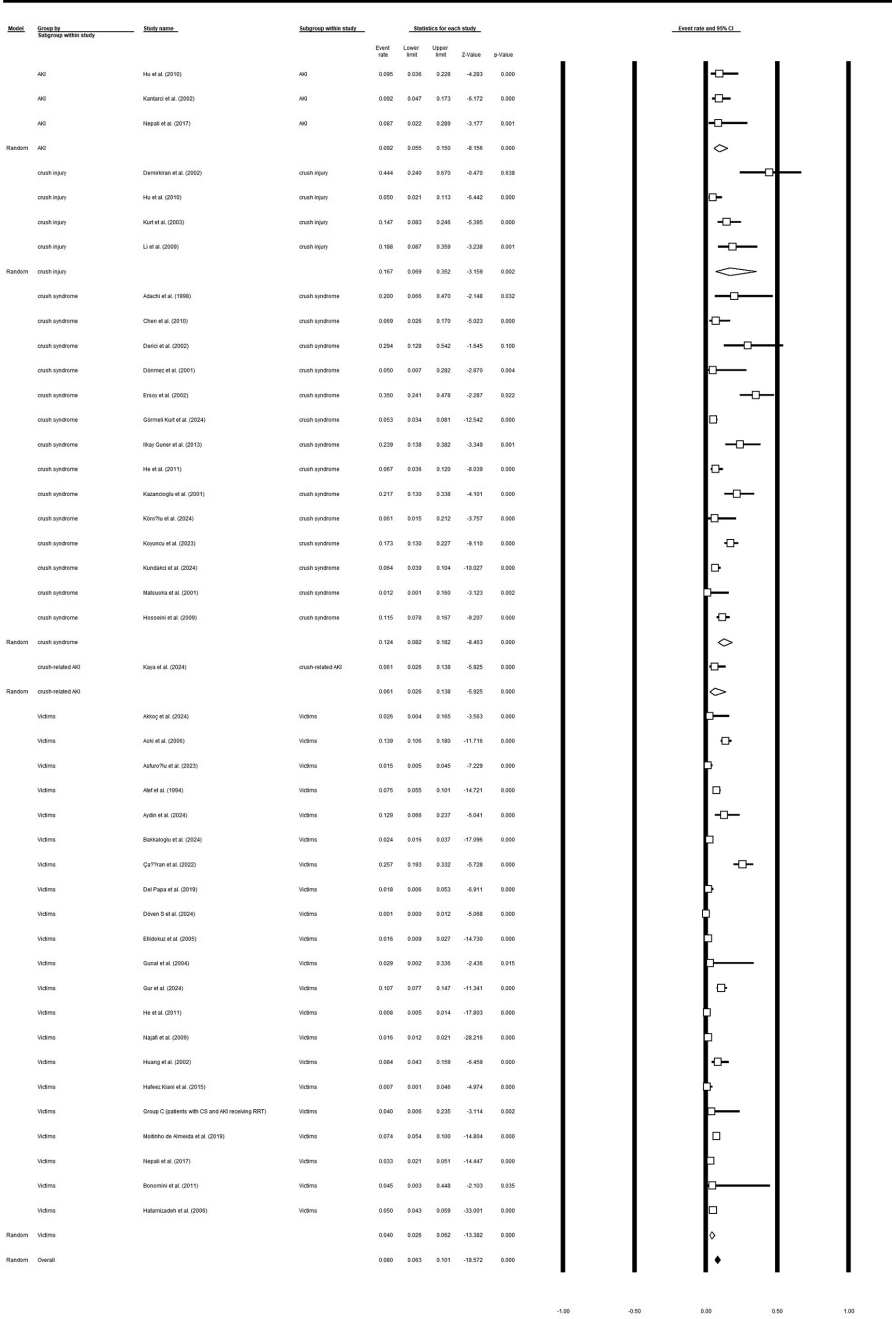
Mortality of affected individuals based on the CS or crush injury or only victims of earthquake

### Meta-analysis of secondary outcomes (clinical sequelae)

One hundred sixty-two study arms reported one or more non-renal sequelae. The overall pooled proportion of any reported sequelae was 0.25 (95% CI 0.23–0.26; Q = 3956.76, df = 161, *p* < 0.001; I² = 95.9%). Prominent pooled estimates included: AKI (32 studies) 0.49 (95% CI 0.38–0.59; I² = 97.7%), AKI within CS (6 studies) 0.65 (95% CI 0.41–0.84; I² = 86.4%), compartment syndrome (5 studies) 0.37 (95% CI 0.26–0.50; I² = 77.6%), disseminated intravascular coagulation (DIC, seven studies) 0.10 (95% CI 0.03–0.27; I² = 90.9%), infections (9 studies) 0.35 (95% CI 0.19–0.54; I² = 91.8%), sepsis (seven studies) 0.11 (95% CI 0.02–0.39; I² = 95.3%), limb/extremity injury (4–5 studies) pooled estimates ~ 0.31–0.50 depending on subgroup. Many individual sequelae showed substantial between-study heterogeneity (Table 4).

**Table 4:** The result of meta-analysis using the CMA software for sequels in patients affected by the earthquake

Groups		Effect size and 95% interval			Test of null (2-Tail)		Heterogeneity			
Group	Number Studies	Point estimate	Lower limit	Upper limit	Z-value	*P*-value	Q-value	df (Q)	*P*-value	I-squared
Abdominal trauma	1	0.14	0.09	0.20	-7.72	0.00	0.00	0	1.00	0.00
Acute HF	1	0.19	0.09	0.36	-3.24	0.00	0.00	0	1.00	0.00
Acute pulmonary edema	1	0.19	0.09	0.36	-3.24	0.00	0.00	0	1.00	0.00
AKI	32	0.49	0.38	0.59	-0.23	0.82	1338.54	31	0.00	97.68
AKI in CS	6	0.65	0.41	0.84	1.25	0.21	36.68	5	0.00	86.37
ARDS	3	0.06	0.01	0.25	-3.28	0.00	13.36	2	0.00	85.03
ARDS	1	0.02	0.00	0.11	-4.04	0.00	0.00	0	1.00	0.00
ARDS in CS	2	0.05	0.02	0.15	-4.82	0.00	4.59	1	0.03	78.21
Bladder rupture	1	0.02	0.00	0.11	-4.04	0.00	0.00	0	1.00	0.00
Cerebral salt wasting/brain edema	1	0.00	0.00	0.01	-6.80	0.00	0.00	0	1.00	0.00
Chest trauma	1	0.34	0.20	0.52	-1.74	0.08	0.00	0	1.00	0.00
CHF in CS	1	0.04	0.02	0.09	-7.61	0.00	0.00	0	1.00	0.00
Compartment syndrome	5	0.37	0.26	0.50	-1.98	0.05	17.82	4	0.00	77.55
Compartment syndrome in CS	3	0.31	0.23	0.41	-3.59	0.00	1.13	2	0.57	0.00
DIC	7	0.10	0.03	0.27	-3.56	0.00	65.80	6	0.00	90.88
DIC in CS	3	0.06	0.02	0.17	-4.61	0.00	8.25	2	0.02	75.76
DVT	1	0.02	0.00	0.11	-4.08	0.00	0.00	0	1.00	0.00
Extremities injury	5	0.48	0.12	0.87	-0.07	0.94	180.50	4	0.00	97.78
Extremity injury in CS	4	0.31	0.11	0.62	-1.20	0.23	27.80	3	0.00	89.21
Fractures	7	0.40	0.23	0.60	-0.94	0.35	145.11	6	0.00	95.87
Hemoglobinuria	1	0.72	0.54	0.85	2.39	0.02	0.00	0	1.00	0.00
Hemothorax	3	0.05	0.01	0.17	-4.26	0.00	7.42	2	0.02	73.06
HTN	1	0.19	0.06	0.45	-2.29	0.02	0.00	0	1.00	0.00
Hypovolemic shock in CS	1	0.10	0.06	0.16	-8.04	0.00	0.00	0	1.00	0.00
Infection	9	0.35	0.19	0.54	-1.59	0.11	97.91	8	0.00	91.83
Infections after 24 h	1	0.20	0.14	0.28	-6.34	0.00	0.00	0	1.00	0.00
Infections within 24 h	1	0.47	0.24	0.71	-0.26	0.80	0.00	0	1.00	0.00
Intestinal injury	1	0.02	0.00	0.11	-4.04	0.00	0.00	0	1.00	0.00
Ischemic encephalopathy	1	0.06	0.01	0.34	-2.62	0.01	0.00	0	1.00	0.00
Limb injuries	4	0.50	0.18	0.82	-0.01	0.99	260.24	3	0.00	98.85
Motor/sensory neuropathy	1	0.33	0.24	0.43	-3.31	0.00	0.00	0	1.00	0.00
Multiple injuries	4	0.25	0.04	0.73	-1.03	0.30	139.94	3	0.00	97.86
Multiple organ failure	4	0.06	0.01	0.29	-2.96	0.00	56.16	3	0.00	94.66
Multiple organ failure in CS	2	0.04	0.02	0.08	-7.79	0.00	0.41	1	0.52	0.00
Musculoskeletal injuries after 24 h	1	0.79	0.71	0.85	6.21	0.00	0.00	0	1.00	0.00
Musculoskeletal injuries within 24 h	1	0.73	0.47	0.90	1.73	0.08	0.00	0	1.00	0.00
Pericardial effusion	2	0.06	0.02	0.16	-5.24	0.00	0.02	1	0.89	0.00
Peripheral neuropathy	2	0.65	0.13	0.96	0.48	0.63	9.15	1	0.00	89.08
Pleural effusion	2	0.05	0.02	0.14	-5.08	0.00	0.04	1	0.84	0.00
Pneumomediastinum	2	0.03	0.00	0.54	-1.87	0.06	38.30	1	0.00	97.39
Pneumonia	1	0.47	0.26	0.70	-0.24	0.81	0.00	0	1.00	0.00
Pneumothorax	2	0.02	0.01	0.09	-5.33	0.00	1.36	1	0.24	26.27
pneumothorax in CS	1	0.08	0.04	0.19	-5.09	0.00	0.00	0	1.00	0.00
Postpyloric ulcus perforation	1	0.02	0.00	0.11	-4.04	0.00	0.00	0	1.00	0.00
Psychiatric problems	1	0.50	0.27	0.73	0.00	1.00	0.00	0	1.00	0.00
Pulmonary embolism	3	0.09	0.01	0.39	-2.42	0.02	7.94	2	0.02	74.83
Pulmonary hemorrhage	1	0.00	0.00	0.01	-6.80	0.00	0.00	0	1.00	0.00
Renal contusion	1	0.02	0.00	0.11	-4.04	0.00	0.00	0	1.00	0.00
Retroperitoneal hematoma	1	0.09	0.03	0.25	-3.74	0.00	0.00	0	1.00	0.00
Rhabdomyolysis	1	0.15	0.09	0.24	-5.78	0.00	0.00	0	1.00	0.00
Sepsis	7	0.11	0.02	0.39	-2.48	0.01	127.79	6	0.00	95.30
Sepsis in CS	1	0.43	0.25	0.64	-0.62	0.53	0.00	0	1.00	0.00
Septic shock	1	0.35	0.25	0.48	-2.25	0.02	0.00	0	1.00	0.00
Soft tissue damage	1	0.04	0.02	0.08	-9.02	0.00	0.00	0	1.00	0.00
Soft tissue injury in CS	2	0.62	0.36	0.83	0.92	0.36	3.56	1	0.06	71.92
Stress ulcer	1	0.06	0.02	0.22	-3.71	0.00	0.00	0	1.00	0.00
Thoracic trauma	3	0.19	0.16	0.21	-17.74	0.00	2.08	2	0.35	3.81
Traumatic shock	1	0.56	0.39	0.72	0.71	0.48	0.00	0	1.00	0.00
URTI	1	0.64	0.54	0.72	2.85	0.00	0.00	0	1.00	0.00
UTI	1	0.13	0.03	0.39	-2.57	0.01	0.00	0	1.00	0.00
Total within							2591.92	102	0.00	
Total between							685.94	59	0.00	
Overall	162	0.25	0.23	0.26	-24.57	0.00	3956.76	161	0.00	95.93

### Mortality rates according to earthquake regions

**Fig. 4:**
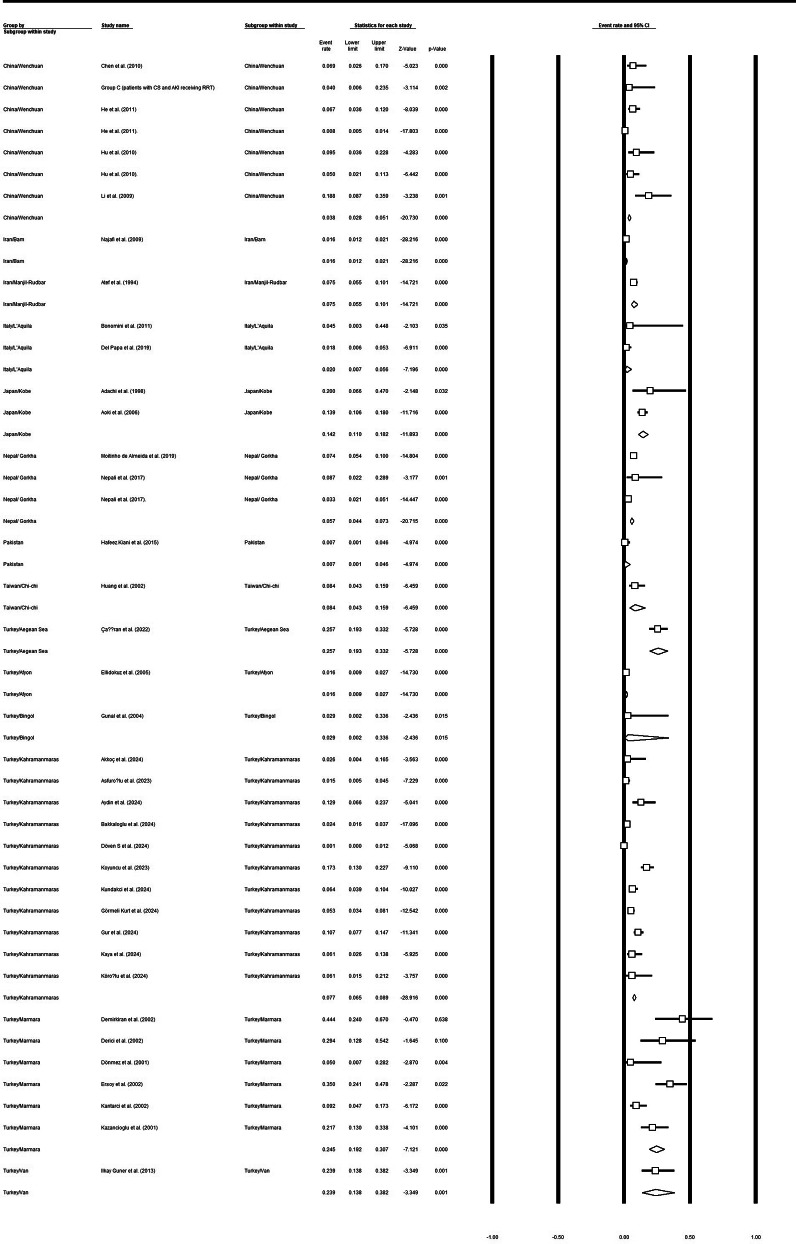
Mortality rate according to the earthquake regions

The meta-analysis of mortality rates stratified by earthquake region demonstrated substantial variability across geographic settings (Table 5; Fig. 4). Pooled mortality estimates ranged from as low as 1% to as high as 26%, reflecting marked regional differences in earthquake impact and contextual factors.

**Table 5:** The result of meta-analysis using the CMA software for mortality according to the regions of the earthquake

Group		Effect size and 95% interval			Test of null (2-Tail)		Heterogeneity			
	Number Studies	Point estimate	Lower limit	Upper limit	Z-value	*P*-value	Q-value	df (Q)	*P*-value	I-squared
China/Wenchuan	7	0.05	0.02	0.14	-5.55	0.00	57.06	6	0.00	89.48
Iran/Bam	1	0.02	0.01	0.02	-28.22	0.00	0.00	0	1.00	0.00
Iran/Manjil-Rudbar	1	0.07	0.05	0.10	-14.72	0.00	0.00	0	1.00	0.00
Italy/L’Aquila	2	0.02	0.01	0.06	-7.20	0.00	0.40	1	0.53	0.00
Japan/Kobe	2	0.14	0.11	0.18	-11.89	0.00	0.43	1	0.51	0.00
Nepal/ Gorkha	3	0.06	0.03	0.10	-8.00	0.00	8.87	2	0.01	77.45
Pakistan	1	0.01	0.00	0.05	-4.97	0.00	0.00	0	1.00	0.00
Taiwan/Chi-chi	1	0.08	0.04	0.16	-6.46	0.00	0.00	0	1.00	0.00
Turkey/Aegean Sea	1	0.26	0.19	0.33	-5.73	0.00	0.00	0	1.00	0.00
Turkey/Afyon	1	0.02	0.01	0.03	-14.73	0.00	0.00	0	1.00	0.00
Turkey/Bingol	1	0.03	0.00	0.34	-2.44	0.01	0.00	0	1.00	0.00
Turkey/Kahramanmaras	11	0.05	0.03	0.09	-9.70	0.00	90.83	10	0.00	88.99
Turkey/Marmara	6	0.23	0.13	0.37	-3.50	0.00	20.59	5	0.00	75.72
Turkey/Van	1	0.24	0.14	0.38	-3.35	0.00	0.00	0	1.00	0.00

In China, analysis of seven studies from the Wenchuan earthquake yielded a pooled mortality rate of 5% (95% CI: 2%–14%), with considerable heterogeneity observed among studies (Q = 57.06, *p* < 0.001; I² = 89.48%). In Iran, the Bam earthquake showed a mortality rate of 2% (95% CI: 1%–2%), while the Manjil–Rudbar earthquake demonstrated a higher mortality rate of 7% (95% CI: 5%–10%); both estimates were derived from single studies and therefore showed no heterogeneity.

European data from Italy’s L’Aquila earthquake, based on two studies, indicated a pooled mortality rate of 2% (95% CI: 1%–6%) with no significant heterogeneity (Q = 0.40, *p* = 0.53; I² = 0%). In Japan, two studies from the Kobe earthquake reported a notably higher pooled mortality rate of 14% (95% CI: 11%–18%), also with negligible heterogeneity (I² = 0%).

In South Asia, the Gorkha earthquake in Nepal, analyzed across three studies, showed a pooled mortality rate of 6% (95% CI: 3%–10%), accompanied by substantial heterogeneity (Q = 8.87, *p* = 0.01; I² = 77.45%). Pakistan, represented by a single study, exhibited a mortality rate of 1% (95% CI: 0%–5%). Similarly, the Chi-Chi earthquake in Taiwan showed a mortality rate of 8% (95% CI: 4%–16%).

Turkey demonstrated a wide variation in mortality across different earthquake events and regions. The Aegean Sea earthquake showed the highest reported mortality rate at 26% (95% CI: 19%–33%), while Marmara earthquakes, based on six studies, yielded a pooled mortality rate of 23% (95% CI: 13%–37%) with substantial heterogeneity (Q = 20.59, *p* < 0.001; I² = 75.72%). The Kahramanmaraş earthquake, analyzed in eleven studies, had a pooled mortality rate of 5% (95% CI: 3%–9%), again with high heterogeneity (Q = 90.83, *p* < 0.001; I² = 88.99%). Lower mortality rates were observed in Afyon (2%; 95% CI: 1%–3%), Bingöl (3%; 95% CI: 0%–34%), and Van (24%; 95% CI: 14%–38%), each derived from a single study.

### Definitions and diagnostic limitations

A major source of heterogeneity was the inconsistent and frequently unreported diagnostic criteria for crush syndrome. In a substantial proportion of studies, diagnostic definitions were recorded as “not mentioned,” precluding meaningful reclassification. Some studies defined CS based on systemic rhabdomyolysis, others on renal failure following crush injury, and some on clinical features of compartment syndrome. This lack of diagnostic granularity limits the reliability of subgroup comparisons between crush injury and crush syndrome and likely inflates between-study heterogeneity.

### Publication bias

Formal assessments of publication bias were not emphasized in the main analysis, as publication bias is less informative for descriptive epidemiologic outcomes where study publication is not primarily driven by statistical significance. These analyses are provided in the Supplementary file 4 for completeness.

### Summary of key clinical findings

Laboratory confirmation of rhabdomyolysis was nearly universal, with peak creatine kinase (CK) levels consistently exceeding 1,000 IU/L across studies. The most frequently reported treatments were intravenous fluid resuscitation, fasciotomy for compartment syndrome, and renal replacement therapy. Detailed management strategies and complication rates from individual studies are synthesized in Tables 2 and 3.

## Discussion

This systematic review confirms that CS remains a leading cause of morbidity and mortality in these settings, with acute AKI representing its most consequential systemic complication. Our pooled estimates indicate significant burdens of dialysis requirement (~ 49%) and a range of serious sequelae. However, substantial heterogeneity underscores the profound influence of variable definitions, disaster contexts, and healthcare response capacities.

The pathophysiological cascade of CS, driven by reperfusion injury and the systemic release of myoglobin and electrolytes, was consistently reflected in the laboratory profiles across studies. Marked elevations in creatine kinase (CK), alongside hyperkalemia, metabolic acidosis, and rising serum creatinine, were near-universal findings [25]. Notably, hyperkalemia emerged as a critical prognostic indicator, strongly associated with both mortality and the need for renal replacement therapy (RRT) [21]. This reinforces its role as a key marker for urgent intervention and risk stratification in mass casualty triage.

A fundamental challenge identified is the lack of a standardized, operational definition for CS across the literature [18]. Definitions varied from isolated limb compression with elevated CK [26] to complex criteria requiring concomitant renal impairment [27] or specific electrolyte imbalances [28]. This diagnostic inconsistency is a primary source of the observed clinical and statistical heterogeneity (I² > 90%), complicating direct comparisons and obscuring the true incidence. The development of consensus diagnostic criteria is therefore a prerequisite for advancing coherent research and clinical protocols.

### Acute Kidney Injury and Renal Replacement Therapy: A Core Challenge

The development of AKI was the predominant complication, with a pooled prevalence of 49%. However, reported rates varied extremely widely (6–100%) [7, 26], a disparity attributable not only to injury severity but also to inconsistent diagnostic criteria (e.g., use of “acute renal failure” vs. modern AKI staging) and profound differences in local healthcare resources and reporting sensitivity [29, 30]. The high pooled proportion of patients requiring dialysis (0.49, 95% CI 0.40–0.58) highlights the acute nephrological demand imposed by such disasters. This demand often collides with devastated infrastructure, including damaged dialysis centers and compromised water and electricity supplies, creating a critical gap in care [31, 32].

The correlation between delayed extrication, higher creatinine levels, and increased RRT need underscores that mortality in CS is often a function of time-sensitive systemic complications rather than the initial trauma itself [8, 27, 33–35]. This evidence strongly supports the prioritization of early, aggressive volume resuscitation, ideally initiated in the pre-hospital setting, to mitigate renal injury [36]. However, the logistics of administering large-volume fluid therapy in a compromised infrastructure remain a significant operational hurdle [17].

### Systemic complications and multidisciplinary management

Beyond renal failure, survivors faced a multitude of systemic and local complications, underscoring the multi-organ nature of CS. Infectious complications, including wound infections and sepsis, were highly prevalent [27, 34, 37–39], reflecting the contaminated disaster environment, open wounds, and frequently delayed definitive surgical care. Pulmonary complications such as ARDS and pleural effusion [8, 40, 41], hematological disturbances like DIC, and neurological sequelae including peripheral nerve injuries [26, 42] were also frequently reported. This spectrum necessitates a coordinated, multidisciplinary approach to critical care, which is exceptionally difficult to implement in overwhelmed and resource-depleted disaster zones [43].

### Management strategies and controversies

The management landscape revealed consistent reliance on aggressive intravenous fluid resuscitation as the cornerstone of preventive therapy [21]. However, specific practices varied. The role of mannitol and alkalinizing agents remains debated, reflected in their inconsistent application across studies [12]. Furthermore, practices such as the use and timing of fasciotomy varied considerably [44]. While often deemed a standard intervention for compartment syndrome [22], its utility in pure crush injury without measurable compartment pressure elevation is controversial [23]. The procedure carries significant risks in disaster settings, including high infection rates and resource-intensive aftercare, highlighting the need for clear, context-specific guidelines [44].

### Determinants of mortality and the imperative of preparedness

Pooled mortality was 8% but varied significantly by subgroup. Mortality was higher among dialysis-dependent patients [45, 46] and was influenced by factors including sepsis, severe hyperkalemia, and delayed fluid therapy [47]. The stark association between delayed extrication and mortality underscores that outcomes are inextricably linked to the efficiency of the entire rescue chain—from community first response to definitive hospital care [35, 36]. This highlights a critical public health imperative: seismic preparedness must extend beyond building codes to include robust emergency medical systems, trained search-and-rescue teams, and pre-established plans for medical surge capacity, particularly in nephrological and critical care [7, 31].

### Risk stratification

Early risk stratification is critical in mass-casualty settings. Validated tools such as the McMahon score [48] and simpler triage systems like the Aoki score [49]have been proposed to predict renal failure and mortality in crush syndrome-related rhabdomyolysis. Incorporation of such tools into disaster response protocols may facilitate timely dialysis prioritization, optimize resource allocation, and improve outcomes in overwhelmed healthcare systems.

### Interpretation amidst heterogeneity

The pooled estimates presented in this meta-analysis must be interpreted in the context of considerable statistical heterogeneity (I² > 90% for most outcomes). This heterogeneity is not merely statistical but reflects profound clinical and methodological diversity across the included studies. Key sources include: (1) the absence of a standardized, operational definition for crush syndrome, leading to inconsistent case ascertainment; (2) variable inclusion criteria, with some studies focusing on AKI populations, others on crush injury, and others on mixed cohorts; (3) differences in disaster settings, rescue capabilities, and healthcare infrastructure across countries and decades; and (4) variability in the timing of outcome assessment relative to the earthquake.

These factors collectively limit the generalizability of any single pooled estimate. Our findings should therefore be viewed as describing a wide *spectrum* of possible outcomes rather than providing precise, universally applicable effect measures. The random-effects model was employed to incorporate this heterogeneity, and subgroup analyses were exploratory. We explicitly caution against overgeneralizing these results to specific future earthquake scenarios without considering local context and medical capacity. The primary value of this synthesis lies in highlighting the consistent, high burden of renal complications and the urgent need for diagnostic standardization to improve future research and preparedness planning.

### Strengths and limitations

A key strength of this review is its comprehensive global scope, encompassing data from over three decades and multiple high-magnitude seismic events. The use of random-effects models and subgroup analyses allowed for the quantification of central tendencies amidst recognized heterogeneity.

However, significant limitations must be acknowledged. The predominance of retrospective observational studies introduces risks of selection bias, incomplete data, and confounding. The high statistical heterogeneity (I² > 90% for most analyses) primarily stems from non-methodological sources: the lack of a standardized, operational definition for CS across the literature, varying diagnostic thresholds for AKI and other complications, and profound contextual differences in earthquake magnitude, rescue infrastructure, and hospital capabilities. Furthermore, publication bias may exist, as studies reporting extreme outcomes or from better-resourced centers might be over-represented. These factors preclude definitive conclusions about causal relationships and complicate direct comparison of outcomes across studies.

We reviewed only studies that evaluated hospitalised patients with crush injury or crush syndrome following earthquakes worldwide. Overall mortality rates after different earthquakes were not assessed in this systematic review. A limited number of studies reported mortality rates among patients with crush syndrome, others focused on patients with crush injury, and some reported mortality only among earthquake victims in general. This heterogeneity makes subgroup analysis more complex and less reliable. In addition, the quality appraisal of earthquake-based retrospective studies is often challenging due to the use of different data sources and methodological approaches [50]. Furthermore, publication bias may exist, as studies reporting extreme outcomes or from better-resourced centers might be over-represented. The frequent lack of reported diagnostic criteria (“NM”) for crush syndrome in many studies further compounds these limitations, adding uncertainty to the true prevalence and potentially biasing subgroup comparisons. These factors preclude definitive conclusions about causal relationships and complicate direct comparison of outcomes across studies.

### Implications for future research and policy

To address these gaps, future investigations should prioritize prospective, registry-based designs to improve data quality. The foremost research priority is to establish and validate internationally accepted diagnostic criteria for CS. Comparative effectiveness research is needed to determine optimal fluid resuscitation strategies and fasciotomy protocols in resource-constrained contexts. Finally, studies evaluating the long-term renal and musculoskeletal outcomes in survivors are essential to understand the full burden of disease. From a policy perspective, the findings argue for the integration of CS management protocols into national disaster response frameworks. This includes stockpiling essential medical supplies, training field personnel in early recognition and treatment, and ensuring regional plans for the rapid deployment of dialysis resources and nephrology expertise.

## Conclusion

This review consolidates evidence that CS is a major determinant of adverse outcomes following earthquakes, characterized by a high incidence of acute kidney injury and significant metabolic derangements. While early fluid resuscitation and renal replacement therapy are established pillars of management, outcomes are heavily modulated by extrication delays and the variable capacity of healthcare systems. The substantial heterogeneity in the literature reflects a lack of standardized definitions and highlights the challenges of disaster medicine research. Moving forward, enhancing preparedness requires a dual focus: the development of consensus-driven clinical guidelines and the strengthening of health systems’ surge capacity, particularly in nephrological care, to mitigate the mortality and morbidity of CS in future seismic disasters. Outcomes in earthquake-related crush syndrome are highly time-dependent. Delayed extrication and delayed initiation of fluid resuscitation are strongly associated with worse renal outcomes and increased mortality. These findings underscore the critical importance of rapid rescue operations, early supportive care, and preparedness planning that prioritizes nephrological surge capacity.

## Supplementary Information

Below is the link to the electronic supplementary material.

Supplementary Material 1

## Data Availability

All the data used in conducting this research is available through the corresponding author upon reasonable request.
