# Supplementary material for: Crush injury syndrome in earthquakes: a systematic review and meta-analysis on its frequency and complications
Source: BMC Emerg Med. 2026 Apr 2;26:140. doi: 10.1186/s12873-026-01516-9 (PMC13169539; doi:10.1186/s12873-026-01516-9)
Supplement: Supplementary file 1 — Supplementary Material 1 [file 12873_2026_1516_MOESM1_ESM.docx]

**Supplementary file 1. PubMed search Strategy**

| Search number | Query | Sort By | Filters |
| --- | --- | --- | --- |
| 3 | #1 AND #2 | |  |
| 2 | ((("Earthquakes") OR ("Disasters")) OR (Disaster*[Title/Abstract])) OR (Earthquake*[Title/Abstract]) | | |
| 1 | (((((((("Crush Injuries") OR ("Compartment Syndromes")) OR ("Rhabdomyolysis")) OR (Crush Injur*[Title/Abstract])) OR (Crush Fracture*[Title/Abstract])) OR (Crush Syndrome*[Title/Abstract])) OR (Rhabdomyolys*[Title/Abstract])) OR (Compartment Syndrome*[Title/Abstract])) OR (Compression Syndrome*[Title/Abstract]) | | |

**Web of Science search Strategy**

| Search number | Query | Sort By | Filters |
| --- | --- | --- | --- |
| 3 | #1 AND #2 | |  |
| 2 | TS=(Disaster* OR Earthquake*) | | |
| 1 | (((((TS=(Crush NEAR/3 Injur*)) OR TS=(Crush NEAR/3 Fracture*)) OR TS=(Crush NEAR/3 Syndrome*)) OR TS=(Rhabdomyolys*)) OR TS=(Compartment NEAR/3 Syndrome*)) OR TS=(Compression NEAR/3 Syndrome* ) | | |

Cinahl search Strategy

| **#** | **Query** |
| --- | --- |
| S9 | S5 AND S8 |
| S8 | S6 OR S7 |
| S7 | (Disaster* OR Earthquake*) |
| S6 | (MH "Disasters+") |
| S5 | S1 OR S2 OR S3 OR S4 |
| S4 | ((Crush N3 Injur*) OR (Crush N3 Fracture*) OR (Crush N3 Syndrome*) OR Rhabdomyolys* OR (Compartment N3 Syndrome*) OR (Compression N3 Syndrome*)) |
| S3 | (MH "Rhabdomyolysis+") |
| S2 | (MH "Compartment Syndromes+") |
| S1 | (MH "Crush Injuries") |

Cochrane library seach Strategy

| ID | Search |
| --- | --- |
| #1 | MeSH descriptor: [Crush Injuries] explode all trees |
| #2 | MeSH descriptor: [Compartment Syndromes] explode all trees |
| #3 | MeSH descriptor: [Rhabdomyolysis] explode all trees |
| #4 | (((crush NEAR/3 injur* ) OR ( crush NEAR/3 fracture* ) OR ( crush NEAR/3 syndrome* ) OR rhabdomyolys* OR ( compartment NEAR/3 syndrome* ) OR ( compression NEAR/3 syndrome*))):ti,ab,kw (Word variations have been searched) |
| #5 | #1 OR #2 OR #3 OR #4 |
| #6 | MeSH descriptor: [Earthquakes] explode all trees |
| #7 | MeSH descriptor: [Disasters] explode all trees |
| #8 | ((disaster* OR earthquake*)):ti,ab,kw (Word variations have been searched) |
| #9 | #6 OR #7 OR #8 |
| #10 | #5 AND #9 |

Medline search Strategy

**Database: Ovid MEDLINE(R) ALL / PubMed(R) <1946 to Present>**
**Search Strategy:**
**1**  exp Crush Injuries/
**2**  exp Compartment Syndromes/
**3**  exp Rhabdomyolysis/
**4**  ((Crush adj3 Injur*) or (Crush adj3 Fracture*) or (Crush adj3 Syndrome*) or Rhabdomyolys* or (Compartment adj3 Syndrome*) or (Compression adj3 Syndrome*)).ab,ti,tw
**5**  1 or 2 or 3 or 4
**6**  exp Earthquakes/
**7**  exp Disasters/
**8**  (Disaster* or Earthquake*).ab,ti,tw.
**9**  6 or 7 or 8
**10**  5 and 9

Scopous search Strategy

( TITLE-ABS-KEY ( ( ( crush W/3 injur* ) OR ( crush W/3 fracture* ) OR ( crush W/3 syndrome* ) OR rhabdomyolys* OR ( compartment W/3 syndrome* ) OR ( compression W/3 syndrome* ) ) ) AND TITLE-ABS-KEY ( ( disaster* OR earthquake* ) ) )

Embase search Strategy

| No. | Query |
| --- | --- |
| #10 | #5 AND #9 |
| #9 | #6 OR #7 OR #8 |
| #8 | disaster*:ab,ti,kw OR earthquake*:ab,ti,kw |
| #7 | 'disaster'/exp |
| #6 | 'earthquake'/exp |
| #5 | #1 OR #2 OR #3 OR #4 |
| #4 | ((crush NEAR/3 injur*):ab,kw,ti) OR ((crush NEAR/3 fracture*):ab,kw,ti) OR ((crush NEAR/3 syndrome*):ab,kw,ti) OR rhabdomyolys*:ab,kw,ti OR ((compartment NEAR/3 syndrome*):ab,kw,ti) OR ((compression NEAR/3 syndrome*):ab,kw,ti) |
| #3 | 'rhabdomyolysis'/exp |
| #2 | 'compartment syndrome'/exp |
| #1 | 'crush trauma'/exp |

| **Supplementary file 2. The result of meta-analysis using the CMA software for Dialysis requirements in patients affected by the earthquake** | | | | | | | | | | |
| --- | --- | --- | --- | --- | --- | --- | --- | --- | --- | --- |
| Group | Number Studies | Effect size and 95% interval | | | Test of null (2-Tail) | | Heterogeneity | | P-value | I-squared |
|  |  | Point estimate | Lower limit | Upper limit | Z-value | P-value | Q-value | df (Q) |  |  |
| AKI | 3 | 0.60 | 0.24 | 0.88 | 0.53 | 0.60 | 8.49 | 2 | 0.01 | 76.45 |
| AKI and CS | 1 | 0.50 | 0.26 | 0.74 | 0.00 | 1.00 | 0.00 | 0 | 1.00 | 0.00 |
| CS | 4 | 0.46 | 0.20 | 0.75 | -0.24 | 0.81 | 14.16 | 3 | 0.00 | 78.81 |
| victims | 30 | 0.49 | 0.38 | 0.59 | -0.27 | 0.78 | 1132.68 | 29 | 0.00 | 97.44 |
| Overall | 38 | 0.49 | 0.40 | 0.58 | -0.19 | 0.85 | 1214.26 | 37 | 0.00 | 96.95 |

| **Supplementary file 3. The result of meta-analysis using the CMA software for Mortality in patients affected by the earthquake** | | | | | | | | | | |
| --- | --- | --- | --- | --- | --- | --- | --- | --- | --- | --- |
| Group | Number Studies | Effect size and 95% interval | | | Test of null (2-Tail) | | Heterogeneity | | P-value | I-squared |
|  |  | Point estimate | Lower limit | Upper limit | Z-value | P-value | Q-value | df (Q) |  |  |
| Mortality |  |  |  |  |  |  |  |  |  |  |
| AKI | 3 | 0.09 | 0.06 | 0.15 | -8.16 | 0.00 | 0.01 | 2 | 0.99 | 0.00 |
| crush injury | 3 | 0.17 | 0.04 | 0.50 | -1.98 | 0.05 | 17.23 | 2 | 0.00 | 88.39 |
| crush syndrome | 12 | 0.12 | 0.07 | 0.18 | -7.54 | 0.00 | 72.47 | 11 | 0.00 | 84.82 |
| crush-related AKI | 1 | 0.06 | 0.03 | 0.14 | -5.92 | 0.00 | 0.00 | 0 | 1.00 | 0.00 |
| Victims | 20 | 0.04 | 0.03 | 0.07 | -11.15 | 0.00 | 370.57 | 19 | 0.00 | 94.87 |
| Overall | 39 | 0.08 | 0.06 | 0.10 | -16.63 | 0.00 | 545.76 | 38 | 0.00 | 93.04 |

Formal tests for small-study effects in mortality meta-analysis did not indicate clear evidence of publication bias: Begg’s rank correlation (Kendall’s τ) was non-significant (two-tailed p = 0.26), and Egger’s regression intercept was not significant (two-tailed p = 0.86) (Supplementary file 4).

Supplementary file 4. Publication bias for mortality outcome
